# Effect of Anti-Inflammatory and Antimicrobial Cosupplementations on Sepsis Prevention in Critically Ill Trauma Patients at High Risk for Sepsis

**DOI:** 10.3389/fphar.2021.792741

**Published:** 2021-11-29

**Authors:** Noha A. Kamel, Moetaza M. Soliman, Maha A. Abo-Zeid, Mona I. Shaaban

**Affiliations:** ^1^ Department of Clinical Pharmacy and Pharmacy Practice, Faculty of Pharmacy, Mansoura University, Mansoura, Egypt; ^2^ Department of Anesthesia, Surgical Intensive Care Unit and Pain Management, Faculty of Medicine, Mansoura University, Mansoura, Egypt; ^3^ Department of Microbiology and Immunology, Faculty of Pharmacy, Mansoura University, Mansoura, Egypt

**Keywords:** vitamin C, vitamin B1, vitamin D, probiotics, leukocyte antisedimentation rate, monocyte chemoattractant protein 1, controlled trial, sepsis

## Abstract

**Background:** Sepsis development in patients with trauma is associated with bad prognosis. This study investigated the effect of immunomodulatory interventions in major trauma patients at high risk for sepsis.

**Methods:** In a randomized, double-blinded, controlled design, severe trauma patients were stratified by leukocyte anti-sedimentation rate (LAR) test into high risk (HR) and low risk (LR) for sepsis. The HR patients were randomly allocated into intravenous vitamin C plus vitamin B1 (HR-CB), intramuscular vitamin D plus oral *Lactobacillus* probiotics (HR-DP), or control (HR-C) groups. The clinical trial was registered at clinicaltrials.gov (https://clinicaltrials.gov/show/NCT04216459).

**Outcomes:** The primary outcome was Acute Physiologic Assessment and Chronic Health Evaluation score II (APACHE II) score. Secondary outcomes included sepsis incidence, changes in Sequential Organ Failure Assessment (SOFA) score, and serum monocyte chemoattractant protein-1 (MCP-1) on day 6 from baseline, 28-day mortality, intensive care unit (ICU), and hospital discharge.

**Results:** The HR-DP, HR-CB, and LR groups showed a significantly lower incidence of sepsis development (20%, 20%, and 16%, respectively, versus 60% in the HR-C group, *p*-value = 0.004). The three groups also showed a significant improvement in APACHE II and SOFA scores. Besides, MCP-1 levels were significantly decreased in HR-DP and HR-CB groups compared to the HR-C group (*p*-value ≤ 0.05). Significantly decreased mortality (10% and 16% versus 60% in the HR-C group) and increased ICU discharge (95% and 84% versus 45% in the HR-C group) were observed in HR-CB and LR groups (*p*-value = 0.001).

**Conclusion:** Both combinations of interventions improved APACHE II scores and reduced sepsis incidence in trauma patients. The LAR combined with injury severity score were good sepsis predictors.

## 1 Introduction

Sepsis is a life-threatening illness associated with poor prognosis (Rudd et al., 2020). Patients with major trauma are prone to septic complications due to the immune dysregulation that occurs after trauma (Hesselink et al., 2019). The incidence of mortality due to post-traumatic sepsis development in the intensive care unit (ICU) is still high (Wafaisade et al., 2011). Both trauma and sepsis cause tissue and cell damages, systemic inflammatory response syndrome, and multiple organ failure in severe cases. The reason for the similarity in body response to trauma and sepsis might be that the antigen structures of mitochondria released during trauma are very similar to the genetic structure of pathogens in sepsis. Nevertheless, the exact underlying mechanisms are not the same (Rozanovic et al., 2016).

The prevention of sepsis in patients with trauma could greatly help avoid the poor prognosis of sepsis and improve patient survival (Ma et al., 2016). The ideal prevention strategy should involve first identifying patients with major trauma at high risk for sepsis who would benefit most from the used immunomodulatory interventions. Early prediction of sepsis development is a key factor that would allow the use of preventive interventions to improve patient prognosis (Jin et al., 2014).

First, the early prediction of sepsis in trauma patients is likely to face many challenges. The surviving sepsis campaign in 2016 defined sepsis as a life-threatening organ dysfunction caused by a dysregulated host response to infection. Organ dysfunction is identified as acute change in the total Sequential Organ Failure Assessment Score (SOFA) score ≥2 points (Singer et al., 2016). On applying sepsis-3 definition in clinical practice, by the time the patient is diagnosed as septic, organ dysfunction has already occurred. Patients with sepsis often have a bad prognosis. Even the survivors suffer from long term physical, psychological, and cognitive disabilities (Sartelli et al., 2018).

Identifying patients at high risk for sepsis before reaching multi-organ failure was never mentioned in the surviving sepsis campaign’s latest guidelines (Singer et al., 2016). On the other hand, relying on the blood culture results to identify patients at high risk for sepsis is not possible either. Infection is rarely confirmed microbiologically. Culture-positive sepsis is observed only in 30%–40% of cases (Singer et al., 2016). The time delay in obtaining culture results and the possibility of false-negative findings limit the usefulness of culture in the early recognition of sepsis (Sweeney et al., 2019).

The limitation of the 2016 sepsis guidelines was addressed in the following 2021 surviving sepsis campaign’s guidelines that recommended implementing sepsis performance improvement programs in healthcare settings. These programs consist mainly of two arms: sepsis screening tools and standard operating procedures. Sepsis screening tools were defined as means of identifying high-risk critically ill patients to allow timely interventions that help improve their prognosis. Standard operating procedures involved usual care by obtaining cultures and administering fluids and antibiotics (Evans et al., 2021). Two methods, leukocyte antisedimentation rate (LAR) and monocyte chemoattractant protein-1 (MCP-1), were used in previous studies for the early prediction of sepsis in trauma and showed positive results (Rozanovic et al., 2016; Wang et al., 2018). The LAR failing to exceed 15% on day 1 (second day from the ICU admission) was used to predict the high risk for sepsis in trauma patients (Rozanovic et al., 2016), whereas the serum MCP-1 levels >240.7 pg/ml on day 0 (within 24 h of admission) was used for the same purpose of identifying patients with trauma at high risk for sepsis development (Wang et al., 2018).

The LAR test offered some advantages that made its use more feasible and affordable in this trial for prediction of sepsis than the MCP-1. These advantages include the performance of LAR using whole blood samples; no storage, preparation, or isolation procedures could cause false activation of leukocytes. Moreover, the LAR test is cheap, reproducible, easy to perform, and time-saving (Bogár et al., 1997). Conversely, the levels of MCP-1 were measured by enzyme-linked immunosorbent assay (ELISA) (Wang et al., 2018). Some disadvantages hinder the clinical use of ELISA in Egyptian ICUs including the tedious and time-consuming procedure besides the necessity for centralized laboratory equipment (Hosseini et al., 2018).

Second, for sepsis prevention in patients with trauma at high risk for sepsis development, using a combination of interventions was recommended. The rationale behind this recommendation was the complex pathophysiology of sepsis involving hundreds of mediators and the failure of previous studies using single intervention targeting a single biomarker (Aird, 2003). Several immunomodulatory interventions have been used in previous studies including intravenous (IV) high-dose vitamin C and vitamin B1, IV stress dose steroids, IV N-acetyl cysteine, intramuscular (IM) or oral high-dose vitamin D, and oral probiotics (Kotzampassi et al., 2006; Bedreag et al., 2015; Sandesc et al., 2018; Tessa et al., 2018; Hasanloei et al., 2020). Positive results were reported including lower incidence of sepsis development and multi-organ dysfunction syndrome with vitamin C and N-acetyl cysteine (Sandesc et al., 2018) and lower peak SOFA scores with vitamin C and vitamin B1 (Tessa et al., 2018). In other contexts, involving the management of sepsis and septic shock in the medical ICU, hydrocortisone, ascorbic acid, and thiamine combination has shown promise (Marik et al., 2017). Vitamin C has antibacterial effects, whereas both vitamin C and vitamin B1 have anti-inflammatory, antioxidant, and mitochondrial protective effects (Marik, 2018). None of the previous studies specifically targeted patients with major trauma at high risk for sepsis.

Vitamin D and probiotics have been used separately in the previous trials focusing on patients with trauma. The reported positive outcomes included reduced incidence of sepsis with synbiotics (Kotzampassi et al., 2006), significantly lower SOFA score, duration of mechanical ventilation, and ICU stay with high-dose oral and IM vitamin D3 (Hasanloei et al., 2020). Vitamin D and probiotics have been used together in contexts other than trauma and have shown a synergistic effect as anti-inflammatory and antimicrobial combination (Abboud et al., 2021).

Previous studies on immunomodulatory interventions in trauma usually monitored the change in interleukin 6 (IL-6) as a proinflammatory cytokine (Kotzampassi et al., 2006; Sandesc et al., 2018; Hasanloei et al., 2020). However, none of the previous studies investigated the effect of immunomodulatory interventions on MCP-1 levels among patients with major trauma. Wang et al. suggested that future studies should investigate their hypothesis that decreasing MCP-1 could confer an associated therapeutic benefit among ICU patients with major trauma (Wang et al., 2018).

Therefore, the aims of the current study were, first, to re-validate LAR as a cheap and available test combined with Injury Severity Score (ISS) to predict the risk for sepsis development in major trauma ICU patients and, second, to investigate the effect of IM vitamin D3 supplementation plus oral probiotics cosupplementation versus IV vitamin C plus vitamin B1 on prevention of sepsis compared to no additional supplementation. This was based on the combined predictable anti-inflammatory and antimicrobial effects of each set of study regimens on sepsis prevention in ICU patients with major trauma at high risk for sepsis development.

## 2 Materials and Methods

### 2.1 Study Design and Location

This was a prospective, randomized, controlled, double-blind study conducted among trauma patients at high risk for sepsis in the ICU. Data were collected from February to November 2020 in the ICUs of Mansoura University Emergency Hospital, Egypt.

### 2.2 Ethics Approval

Study procedures complied with the 1964 Declaration of Helsinki and its later amendments (Rickham, 1964; Baker, 2020). Confidentiality of patient data was preserved. No patient identifiers were used in the datasheet. The study was approved by the Institutional Review Board (IRB), Faculty of Medicine (IRB # R.19.12.707) and Research Ethics Committee, Faculty of Pharmacy, Mansoura University. Informed consent was obtained from all patients or their relatives in case the patient was unable to provide consent. The clinical trial had been registered at clinicaltrials.gov (https://clinicaltrials.gov/show/NCT04216459).

### 2.3 Inclusion and Exclusion Criteria

Inclusion criteria consisted of admission to ICU within 24 h from trauma onset with ISS ≥ 16 and age ≥ 18 years. The exclusion criteria included pregnant or breastfeeding women and immune deficient patients or patients receiving immunosuppressant drugs. Patients at high risk for sepsis (LAR < 15%) who had serum vitamin D level <10 ng/ml or >30 ng/ml or serum calcium level >10 mg/dl were excluded. Besides, patients with a history of primary parathyroid disease and those with contraindications to enteral administration were also excluded. Patients with end-stage renal disease on renal replacement therapy were not eligible for the study. Moreover, patients with oxalate nephropathy or glucose-6 phosphate dehydrogenase deficiency were also not eligible for the study.

### 2.4 Outcomes

The primary outcome of the study was the change in Acute Physiologic Assessment and Chronic Health Evaluation score II (APACHE II) score defined as day 6 minus day 0 score, while the secondary outcomes included the change in SOFA score and MCP-1 in addition to number of patients who developed sepsis within the first week. Blood cultures were used as a possible documentation for infection. Moreover, C-reactive protein (CRP) level and erythrocyte sedimentation rate (ESR) were also measured on days 0 and 6 for all included patients. Additional secondary outcomes included ICU discharge, hospital discharge, and mortality within 28 days for all patients.

In a secondary analysis, the predictive value of LAR combined with ISS to predict the risk for sepsis development in severe trauma ICU patients was evaluated.

### 2.5 Sample Size

Sample size calculation was based on APACHE II scores achieved after receiving vitamin C, vitamin D, and probiotics in previous studies (Sanaie et al., 2014; Atalan and Güçyetmez, 2017; Sandesc et al., 2018; Hasanloei et al., 2020). For vitamin C, the mean ± standard deviation (SD) APACHE II score was 8.00 ± 0.99 in the treated group versus 10.50 ± 2.10 in the control group (Sandesc et al., 2018). The estimated mean ± SD APACHE II score after receiving IM vitamin D injection was 9.30 ± 0.95 compared to 10.20 ± 0.50 in the placebo arm (Atalan and Güçyetmez, 2017; Hasanloei et al., 2020). For probiotics, the mean APACHE II score was 13.85 ± 4.82 in patients treated with probiotics versus 20.85 ± 7.55 in the control arm (Sanaie et al., 2014).

G*Power version 3.0.10 was used for sample size calculation. The *t*-test was used to detect difference between two independent means (two groups), two-tailed, with *α* error = 0.05 and power = 89%. The effect sizes were 1.5228481, 1.1855969, and 1.1051758, whereas the total calculated sample sizes were 10, 16, and 18 patients in each arm for vitamin C, vitamin D, and probiotics, respectively. To overlap the probable dropout of patients, 10% of the calculated sizes were added, making the total calculated sample sizes of 11, 18, and 20 for vitamin C, vitamin D, and probiotics, respectively. Thus, we decided to include 20 patients in each group.

### 2.6 Patient Allocation

After ICU admission of patients with ISS ≥ 16, all patients were evaluated. The included patients with high risk for sepsis (LAR < 15%) were randomly allocated, at 1:1:1 ratio, into one of three groups each consisting of 20 patients, using sealed opaque envelopes. Patients in the first group did not receive any additional supplement and represented the control group (HR-C group). Patients in the second group received vitamin D plus probiotics (HR-DP group), while patients in the third group received vitamin C plus vitamin B1 (HR-CB group). The low-risk (LR) group (LAR ≥ 15%) did not receive any special therapy.

### 2.7 Clinical Data Collection

#### 2.7.1 Baseline Characteristics

Demographic characteristics (age, sex, weight, and height), comorbidities, initial ventilatory status, Glasgow coma score (GCS), and laboratory values were collected on admission. The ISS determination was performed according to Baker et al. (1974). Abbreviated injury scale for each type of injury in different body regions was determined according to chart for clinical use (Civil and Schwab, 1988).

On day 0, recordings of APACHE II (Knaus et al., 1985) and SOFA (Vincent et al., 1996) scores were conducted for all included patients. Then, 3 cm of blood sample was drawn within 24 h of ICU admission for measurement of MCP-1. Besides, ESR and CRP levels were measured initially on day 0. After that, on day 1, peripheral venous blood samples were collected for determination of LAR and serum 25-hydroxyvitamin D levels.

#### 2.7.2 Medications Used in the Intervention Groups

In the HR-DP group, patients received vitamin D as one IM injection (400,000 IU of vitamin D3; two ampoules of Devarol-S®, Memphis Co. for Pharmaceutical and Chemical Industries, Egypt) on day 1 in addition to *Lactobacillus* probiotics (Lacteol Fort ® 10 billion colony-forming unit sachets, manufactured by Rameda Pharmaceutical Company under license of Axcan Pharma S.A, France) in a dose of six sachets (one pack) twice a day (at 9 a.m. and 9 p.m.) orally (either directly or through Ryle’s tube feeding) starting from day 1 for 48 h.

Patients of the HR-CB group received from day 1 a dose of 1 g of vitamin C (one ampoule of Wörwag Pharma GmbH and Co. KG® Vitamin C 1000 mg) plus 200 mg of vitamin B1 (two ampoules of Pascoe pharmaceutical preparations GmbH® vitamin B1 100 mg). Vitamin C plus vitamin B1 were infused intravenously in 500 ml of saline over 30 min four times at 12-h intervals for 48 h.

Intradermal skin testing (IDT) for vitamin B1 hypersensitivity was conducted in patients of the HR-CB group with unspecified history of allergy to vitamin B1. Patients showing allergy to vitamin B1 were excluded from the study. Blood gases were investigated for metabolic acidosis. Patients in the HR-CB group showing metabolic acidosis on day 1 were also excluded from the study.

The investigator who knew the allocation of groups and was responsible for the drug administration was excluded in all data collection.

#### 2.7.3 Patient Follow up

On day 6, SOFA and APACHE II scores were recorded for all groups. Moreover, a blood sample was obtained from all patients for determination of MCP-1 (in HR groups), ESR, and CRP level (in all groups) measurements. Changes in SOFA and MCP-1 were defined as day 6 minus initial (day 0) values. For LR group, outcomes were the same as the other three groups except for change in MCP-1 as MCP-1 for this group was only measured on day 0. Eight centimeters of blood were collected for aerobic blood culture (30-ml bottle manufactured by Zhuhai DL Biotech Co., Ltd., China).

During the whole ICU admission, all patients in the four groups were carefully monitored and managed according to the ICU protocol. The number of patients who developed sepsis in each group within 7 days was recorded. Sepsis development within 7 days was confirmed according to the sepsis-3 criteria (Singer et al., 2016). Sepsis was assigned if there was an increase in patient’s SOFA score by two or more points in addition to suspected or documented source of infection (Singer et al., 2016). Furthermore, the duration of mechanical ventilation for patients who needed mechanical ventilation from day 0 in each group was observed by the end of the first week. All included patients were followed for ICU discharge and hospital discharge within 28 days. Also, ICU mortality and hospital mortality (including patients who died in the ICU or after discharge from it in the ward) within 28 days were recorded.

For fear that the patient cannot complete the study (due to transfer outside hospital or death), after completion of study treatment regimen, and before the patient completes day 6, a reserved blood sample and blood culture were collected on day 3. This reserved blood sample was used for MCP-1 (in HR groups), ESR, and CRP level measurements. These reserved samples and blood culture taken on day 3 were collected to be analyzed immediately (except for MCP-1), recorded if the patient did not complete the study, and discarded if day 6 blood sample and blood culture were collected. Also, for those patients, the last recorded APACHE II and SOFA scores (after day 2) were forwarded for assessment, whereas if a patient was discharged to the ward before day 6 but after completing the study regimen in the ICU, the last recorded APACHE II score in the ICU just before discharge was used. Then, the patient was followed in the ward, and the final SOFA score, ESR, CRP level, MCP-1 level (if HR group), and blood culture were collected in the ward on day 6. Patients who were unable to complete their study treatment regimens in the ICU due to very early discharge or death were excluded from the study.

#### 2.7.4 Monitoring of Adverse Events

Serum creatinine level in the ICU was routinely monitored for any significant elevations. Moreover, the serum creatinine level on day 6 was compared to day 0 to record the occurrence of acute kidney injury (AKI). Patients with AKI were managed according to the Kidney disease Improving Global Outcomes (KDIGO) guidelines (Khwaja, 2012).

#### 2.7.5 Details of the Performed Measurements

##### 2.7.5.1 Serum 25-Hydroxyvitamin D Measurement

Serum vitamin D level was assessed using the LIAISON® analyzer, DiaSorin S.p.A. The LIAISON® 25-hydroxyvitamin D assay is a direct, competitive chemiluminescent immunoassay for quantitative determination of total 25-hydroxyvitamin D in serum or plasma. This method of immunoassay is FDA approved (FDA, 2007).

##### 2.7.5.2 MCP-1 Measurement

Blood samples (3 cm) were obtained in vacuum red cap disposable plain blood tubes (GD050A, Gong Dong, China) and centrifuged at 370 × g for 5 min (Centrifuge, Sigma, Germany, model 2-16P). Serum samples were collected and stored at −80°C, analyzed together after all patient enrollments. The MCP-1 was assessed using the commercially available Invitrogen Human C-C motif chemokine ligand 2 [CCL2 (MCP-1)] ELISA kit (Thermo Fisher Scientific, Catalog Number BMS281).

Two sets of the ELISA kits were used according to the manufacturer’s instructions. Each kit contained one plate [Microwell Plate (12 strips of eight wells each) coated with monoclonal antibody to human MCP-1]. Samples were diluted at 1:5 [20 μl sample +80 μl assay buffer (1×)]. The standard curve was constructed, and the MCP-1 level in each sample was retrieved from the standard curve and multiplied by the dilution factor (×5). Samples exceeding standard concentration were further externally prediluted.

##### 2.7.5.3 ESR and CRP Measurement

The ESR was measured by modified Westergren method using Streck® ESR-10 Manual Rack for the Modified Westergren Sed Rate, Streck® 240321. However, the CRP level was measured by nephelometry using the BN™ II System nephelometric analyzer.

##### 2.7.5.4 LAR Measurement

Peripheral venous blood (1.28 ml) was collected in sodium citrate anticoagulated tube (vacuum blood tube containing buffered sodium citrate solution with a concentration of 3.8%, 8 × 120 mm, 1.28 ml, GD0128ESR, Gong Dong, China). After 1 h of blood sedimentation, using an automatic cell counter (Mindray BC-2800 Auto Hematology Analyzer), leukocyte count in the upper (U) and lower (L) half of blood column was determined. LAR was calculated according to the equation described by Rozanovic et al. (2016): $LAR=\frac{U-L}{U+L}\times 100$ .

### 2.8 Statistical Analysis

The IBM® SPSS® 26.0.0 statistical software was used to perform statistical analyses. Shapiro–Wilk test for normality was performed. Quantitative data were summarized as mean ± SD or median, interquartile range according to normality. Qualitative data were summarized as frequency (percentage). To detect differences between groups, analysis of variance (ANOVA), Kruskal–Wallis, and chi-square tests were used for parametric, nonparametric, and categorical variables, respectively. If significant differences between groups were found, appropriate post-hoc tests were performed. Post-hoc tests after ANOVA were determined according to homogeneity of variances. Dunn’s and Monte Carlo post-hoc tests were conducted after Kruskal–Wallis and chi-square tests, respectively.

To determine if there were significant differences between day 0 and 6 scores (SOFA or APACHE II) within the same group, paired *t*-test and Wilcoxon signed-rank test were used for parametric and nonparametric data, respectively.

Kaplan–Meier and log rank test were used to compare ICU mortality between HR groups. Cox’s proportional hazards model was used to identify significant independent predictors associated with ICU mortality with calculation of the hazard ratios and 95% confidence intervals. Univariate models were used for determining which variables could be associated with ICU mortality in HR groups (60 patients). The tested variables in the univariate model included the effect of study treatment (CB and DP interventions compared to no intervention in the HR-C group), the initial GCS (three to eight versus higher GCS), the need for vasopressors, ISS (≥25 versus lower ISS), sepsis development (by the end of the first week), and needing mechanical ventilation at admission. Only variables that showed statistical significance in univariate models were included in the multivariate model.

Receiver operating characteristics (ROC) curve was used to evaluate the predictive ability of different sepsis predictors (MCP-1, ISS, 100-LAR, and combinations of 100-LAR + ISS or MCP-1+ISS) among the HR-C and LR groups. Test performance for predictors was evaluated as failed (AUC, 0.5–0.6), poor (AUC, 0.6–0.7), fair (AUC, 0.7–0.8), good (AUC, 0.8–0.9), and excellent (0.9–1) (Hosmer et al., 2013). Probability value (*p*-value) ≤ 0.05 was considered statistically significant.

## 3 Results

**FIGURE 1:**
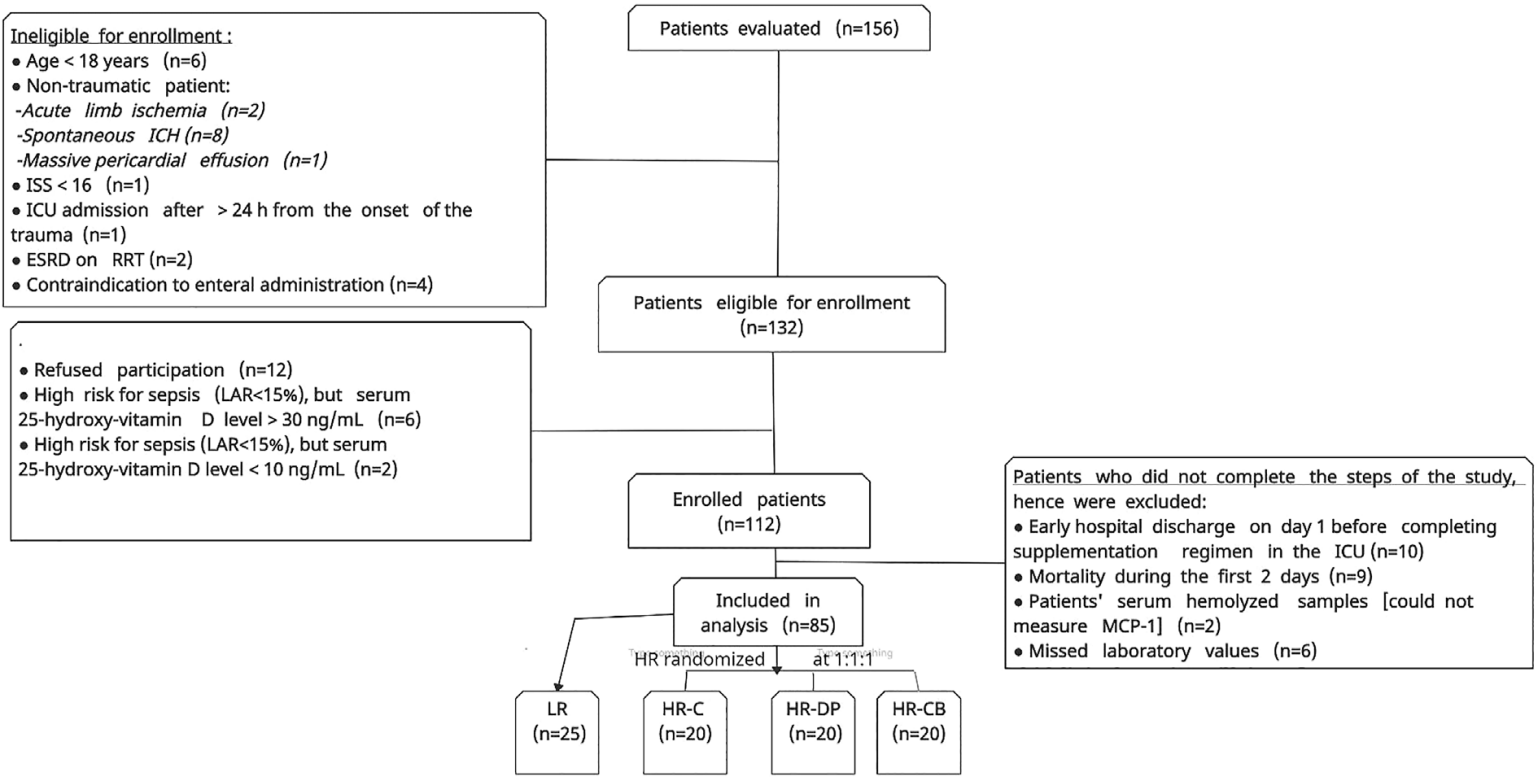
Flow chart of patient enrollment process. ICH: intracerebral hemorrhage, ISS: injury severity score, ESRD: end-stage renal disease, RRT: renal replacement therapy, LR: low risk for sepsis group, HR-C: high risk for sepsis control group, HR-DP: high risk for sepsis vitamin D and probiotics group, HR-CB: high risk for sepsis vitamin C and vitamin B1 group, MCP-1: monocyte chemoattractant protein-1. LAR: leukocyte anti-sedimentation rate.

Between February and November 2020, 156 patients were evaluated. After ruling out patients who did not fulfill the study criteria, 112 patients were enrolled after obtaining informed consent. Then, 27 patients were excluded as they did not complete the steps of the study. Afterward, 85 patients had successfully completed the study (Figure 1).

### 3.1 Patients’ Demographics, Initial Ventilatory Status, and Basal Lab Values

Patients’ demographic data and initial ventilatory status (Table 1) showed no statistically significant difference between groups. Table 2 demonstrates the basal laboratory values. The highest value of LAR was found in the LR group, showing significant increase when compared to the other three groups (*p*-value < 0.0001). Similarly, arterial oxygen saturation and serum 25-hydroxyvitamin D level were significantly high in the LR group compared to high-risk groups with *p*-values of 0.002 and <0.0001, respectively.

**TABLE 1:** Patients’ demographics and initial ventilatory status on admission in each group.

Characteristic	LR (*n* = 25)	HR-C (*n* = 20)	HR-DP (*n* = 20)	HR-CB (*n* = 20)	*p*-value
Age (years)	42.52 ± 18.84	48.75 ± 19.65	44.95 ± 17.54	42.15 ± 15.90	0.63^a^
Male/female number (%)	23 (92%)/2 (8%)	19 (95%)/1 (5%)	17 (85%)/3 (15%)	16 (80%)/4 (20%)	0.47^b^
Height (cm)	165.00 (160.00,170.00)	167.50 (161.25,173.00)	167.50 (160.00,173.00)	165.00 (160.00,171.50)	0.65^c^
Weight (kg)	74.72 ± 7.00	74.50 ± 7.24	74.50 ± 6.67	72.00 ± 7.33	0.56^a^
Comorbidities					
No comorbidities	19 (70.4%)	13 (52%)	13 (54.2%)	17 (77.3%)	0.83^b^
Hypertension	2 (7.4%)	4 (16%)	4 (16.7%)	2 (9.1%)	
Diabetes mellitus	2 (7.4%)	4 (16%)	4 (16.7%)	2 (9.1%)	
Chronic liver disease	2 (7.4%)	2 (8%)	1 (4.2%)	1 (4.5%)	
History of cerebral stroke	2 (7.4%)	0 (0%)	1 (4.2%)	0 (0%)	
Other comorbidities	0 (0%)	2 (8%)	1 (4.2%)	0 (0%)	
Ventilatory status on day 0					0.49^b^
On room air	13 (52%)	5 (25%)	6 (30%)	12 (60%)	
On nasal cannula	2 (8%)	1 (5%)	1 (5%)	1 (5%)	
On Oxygen mask	5 (20%)	5 (25%)	5 (25%)	3 (15%)	
On Mechanical ventilation	5 (20%)	9 (45%)	8 (40%)	4 (20%)	

Data are mean ± standard deviation, median (interquartile range) or number (incidence). LR: low risk for sepsis group, HR-C: high risk for sepsis control group, HR-DP: high risk for sepsis vitamin D and probiotics group, HR-CB: high risk for sepsis vitamin C and vitamin B1 group.

a : Analysis of variance (ANOVA) used to detect differences among groups.

b : Monte Carlo test with 95% confidence interval used to detect differences among groups.

c : Kruskal–Wallis test used to detect differences among groups.

**TABLE 2:** Basal laboratory values.

Laboratory test	LR (*n* = 25)	HR-C (*n* = 20)	HR-DP (*n* = 20)	HR-CB (*n* = 20)	*p*-value
Day 0					
Virology					
HBV positive	0 (0%)	2 (10%)	0 (0%)	2 (10%)	0.24^e^
HCV positive	7 (28%)	6 (30%)	3 (15%)	3 (15%)	0.58^e^
Hemoglobin (g/dl)	12.1 (10.40, 12.75)	12.15 (10.20, 13.8)	11.25 (9.60, 13.05)	11.55 (10.03, 13.43)	0.73^c^
MCHC (g/dl)	33 (32.10, 33.85)	32.30 (31.20, 33.25)	33.05 (32.03, 33.30)	33.25 (32.30, 33.78)	0.064^c^
Prothrombin time (seconds)	15 (14.35, 15.80)	15.55 (14.63, 16.50)	14.85 (13.90, 16.08)	15.35 (14.80, 16.20)	0.47^c^
INR	1.15 (1.08, 1.28)	1.20 (1.10, 1.32)	1.13 (1.03, 1.25)	1.2 (1.13, 1.30)	0.41^c^
Lymphocyte %	8.1 (6.95, 11.95)	8.45 (6.30, 12.00)	8.25 (6.70, 11.73)	9.35 (6.63, 13.05)	0.96^c^
ALT (U/L)	28 (25.00, 44.1)	44 (29.75, 68.75)	32 (27.00, 66.25)	26 (21.50, 75.50)	0.31^c^
Albumin (g/dl)	3.67 ± 0.67	3.52 ± 0.68	3.55 ± 0.72	3.83 ± 0.66	0.47^a^
Serum calcium (mg/dl)	7.55 ± 0.94	7.88 ± 0.76	7.74 ± 0.95	7.86 ± 0.92	0.59^a^
RBG (mg/dl)	163.00 (140.00,187.00)	180.00 (152.50,252.00)	187.50 (143.50,239.00)	168.50 (154.00,211.00)	0.29^c^
Blood gases					
PH	7.39 (7.37, 7.43)	7.37 (7.31, 7.40)	7.37 (7.33, 7.42)	7.37 (7.31, 7.40)	0.059^c^
PaCO_2_ (mmHg)	35.26 ± 6.08	34.73 ± 8.42	33.29 ± 7.58	34.47 ± 5.63	0.82^a^
SaO_2_ (%)	97.60 (93.00,100.00)	87.35 (64.28, 95.93)^f^	80.40 (62.43, 93.10)^f^	93.35 (65.78, 97.50)^f^	<0.0001^d^
Serum 25-hydroxy vitamin D level (ng/ml)	32.30 (25.05, 36.91)	16.00 (12.22, 21.63)^f^	16.22 (12.12, 23.32)^f^	20.50 (17.50, 23.00)^f^	<0.0001^d^
Day 1					
LAR (%)	34.15 ± 9.18	5.60 ± 3.17^f^	6.00 ± 2.71^f^	6.68 ± 4.28^f^	<0.0001^b^

Data are mean ± standard deviation, median (interquartile range) or number (incidence). LR: low risk for sepsis group, HR-C: high risk for sepsis control group, HR-DP: high risk for sepsis vitamin D and probiotics group, HR-CB: high risk for sepsis vitamin C and vitamin B1 group. MCHC: mean corpuscular hemoglobin concentration, INR: international normalized ratio, ALT: alanine aminotransferase, HBV: hepatitis B virus, HCV: hepatitis C virus, PaCO_2_: arterial partial pressure of carbon dioxide, SaO_2_: arterial oxygen saturation, LAR: leukocyte anti-sedimentation rate, RBG: random blood glucose.

a : Analysis of variance (ANOVA) used to detect differences among groups.

b : Analysis of variance (ANOVA) followed by post-hoc test according to homogeneity of variances (Levine’s test), where we used Bonferroni post-hoc test if equal variances were assumed and Games-Howell post-hoc test if no homogeneity of variances was found. For both Bonferroni and Games-Howell post-hoc tests, the mean difference was significant at the 0.05 level.

c : Kruskal–Wallis test used to detect differences among groups.

d : Kruskal–Wallis test followed by post-hoc test (Dunn’s test).

e : Monte Carlo test with 95% confidence interval used to detect differences among groups.

f = significant with LR group. Significance level at *p*-value ≤ 0.05.

### 3.2 Injury Characteristics of Each Group

There were no significant differences between the groups with respect to ISS, cause of trauma, and primary diagnosis (type of trauma), even in the segmental injury description. Intracranial hematoma (≤100 ml or unspecified) represented the most prevalent injury in all patient groups either isolated or combined with other traumas (Tables 3, 4).

**TABLE 3:** Injury characteristics of patients in each group.

	LR (*n* = 25)	HR-C (*n* = 20)	HR-DP (*n* = 20)	HR-CB (*n* = 20)	*p*-value
ISS	16 (16, 21)	20 (16, 25)	19 (16, 24.25)	21 (17, 28.5)	0.07^a^
Cause of trauma					
Road traffic accident	15 (60%)	11 (55%)	15 (75%)	14 (70%)	0.32^b^
Stab (abdomen or chest)	2 (8%)	1 (5%)	0 (0%)	1 (5%)	
Fall injury (from height, to the ground or downstairs)	8 (32%)	8 (40%)	5 (25%)	5 (25%)	
Primary diagnosis					
Multiple trauma	11 (44%)	14 (70%)	12 (60%)	16 (80%)	0.24^b^
Head trauma	8 (32%)	3 (15%)	6 (30%)	3 (15%)	
Spine trauma	0 (0%)	1 (5%)	0 (0%)	0 (0%)	
Extremity trauma	2 (8%)	0 (0%)	0 (0%)	1 (5%)	
Thoracic trauma	1 (4%)	2 (10%)	1 (5%)	0 (0%)	
Abdominal trauma	3 (12%)	0 (0%)	1 (5%)	0 (0%)	

Data are median (interquartile range) or number (incidence). LR: low risk for sepsis group, HR-C: high risk for sepsis control group, HR-DP: high risk for sepsis vitamin D and probiotics group, HR-CB: high risk for sepsis vitamin C and vitamin B1 group. ISS: injury severity score.

a : Kruskal–Wallis test used to detect differences among groups.

b : Monte Carlo test with 95% confidence interval used to detect differences among groups.

**TABLE 4:** Segmental injury descriptions in study groups.

	LR (*n* = 25)	HR-C (*n* = 20)	HR-DP (*n* = 20)	HR-CB (*n* = 20)	*p*-value
Hematoma epidural, subdural or intracranial ≤ 100 ml or unspecified	10 (20.8%)	7 (16.7%)	10 (25.6%)	11 (20.4%)	0.95^a^
Traumatic subarachnoid hemorrhage	2 (4.2%)	1 (2.4%)	1 (2.6%)	2 (3.7%)	
Traumatic cerebral edema	2 (4.2%)	2 (4.8%)	2 (5.1%)	3 (5.6%)	
Fracture base without CSF leak	3 (6.3%)	2 (4.8%)	2 (5.1%)	3 (5.6%)	
Fracture orbit, maxilla or zygoma (unspecified)	1 (2.1%)	0 (0%)	3 (7.7%)	1 (1.9%)	
Fracture orbit open or displaced	1 (2.1%)	1 (2.4%)	1 (2.6%)	0 (0%)	
Cervical cord lesion (incomplete) with preservation of significant sensation	0 (0%)	1 (2.4%)	1 (2.6%)	1 (1.9%)	
Dislocation or fracture of thoracic or lumbar spine (unspecified)	0 (0%)	1 (2.4%)	0 (0%)	0 (0%)	
Dislocation of lamina, body, facet, or pedicle of thoracic spine	1 (2.1%)	0 (0%)	0 (0%)	0 (0%)	
Fracture radius, ulna, clavicle, scapula, tibia, fibula, or tarsals	4 (8.3%)	5 (11.9%)	4 (10.3%)	9 (16.7%)	
Fracture tibia, radius, or ulna open or displaced	4 (8.3%)	2 (4.8%)	1 (2.6%)	3 (5.6%)	
Sprain or contusion wrist	3 (6.3%)	4 (9.5%)	0 (0%)	3 (5.6%)	
Fracture femur (open)	1 (2.1%)	1 (2.4%)	0 (0%)	0 (0%)	
Traumatic above knee amputation	1 (2.1%)	0 (0%)	0 (0%)	1 (1.9%)	
Multi-lobar lung contusion	3 (6.3%)	5 (11.9%)	5 (12.8%)	2 (3.7%)	
Lung contusion< 1 lobe	5 (10.4%)	2 (4.8%)	2 (5.1%)	3 (5.6%)	
Bilateral hemothorax	1 (2.1%)	3 (7.1%)	0 (0%)	1 (1.9%)	
Bilateral pneumothorax	1 (2.1%)	3 (7.1%)	1 (2.6%)	3 (5.6%)	
Unilateral pneumothorax	0 (0%)	0 (0%)	1 (2.6%)	2 (3.7%)	
Unilateral hemothorax	0 (0%)	0 (0%)	1 (2.6%)	3 (5.6%)	
Rib fracture with pneumothorax	0 (0%)	1 (2.4%)	1 (2.6%)	2 (3.7%)	
Superficial or unspecified laceration of duodenum, ileum, or liver	2 (4.2%)	0 (0%)	0 (0%)	1 (1.9%)	
Grade III splenic hematoma	2 (4.2%)	0 (0%)	1 (2.6%)	0 (0%)	
Retroperitoneal hematoma, symphysis pubis separation	1 (2.1%)	0 (0%)	1 (2.6%)	0 (0%)	
Minor contusion kidney	0 (0%)	1 (2.4%)	1 (2.6%)	0 (0%)	

Data are number (incidence). LR: low risk for sepsis group, HR-C: high risk for sepsis control group, HR-DP: high risk for sepsis vitamin D and probiotics group, HR-CB: high risk for sepsis vitamin C and vitamin B1 group. CSF: cerebrospinal fluid.

a : Monte Carlo test with 95% confidence interval used to detect differences among groups.

### 3.3 Change in Inflammatory Indices on Day 6 Compared to Day 0

Table 5 shows the serum levels of the investigated inflammatory indices on day 0 and 6 in each group. The MCP-1 level was significantly high on day 0 in HR groups compared to the LR group (*p*-value < 0.0001). On day 6, a significant decrease was detected in both HR-CB and HR-DP groups compared to the HR-C group (*p*-value = 0.006).

**TABLE 5:** Serum levels of inflammatory indices among groups on day 0 and day 6.

Serum level	Day	LR (*n* = 25)	HR-C (*n* = 20)	HR-DP (*n* = 20)	HR-CB (*n* = 20)	*p*-value
MCP-1 (pg/ml)	Day 0	89.26 (57.29,133.97)	193.07 (118.67,427.23)^a^	320.15 (172.86,493.62)^a^	351.82 (179.37,759.99)^a^	<0.0001^h^
	Day 6	—	247.56 (191.15, 503.30)^d^	151.83 (81.50, 274.13)^b^ ^,^ ^d^	144.79 (82.94, 187.12)^b^ ^,^ ^d^	0.006^h^
—	Delta MCP-1	—	44.82 (17.49, 119.67)	−113.61 (−283.54, −76.62)^b^	−219.30 (−494.19, −109.43)^b^	<0.0001^h^
ESR 1st hour (mm/h)	Day 0	32.00 (16.00, 50.00)	24.00 (10.00, 50.00)	26.00 (9.00, 39.00)	29.00 (19.25, 62.25)	0.47^g^
	Day 6	31.00 (15.00, 57.50)	53.50 (27.50, 87.50)^d^	37.50 (20, 63.75)^d^	46.50 (26.25, 58.75)	0.15^g^
—	Delta ESR 1st hour	1.56 ± 19.87^b^	24.25 ± 24.28	14.65 ± 29.70	4.75 ± 26.65	0.02^f^
ESR 2nd hour (mm/h)	Day 0	64.16 ± 38.04	55.15 ± 39.55	51.20 ± 30.56	68.65 ± 38.54	0.41^e^
	Day 6	60.00 (30.00,100.00)	85.00 (57.50,120.00)^d^	82.50 (41.25,107.50)^d^	79.50 (52.50, 99.50)	0.13^g^
—	Delta ESR 2nd hour	−1.16 ± 38.80^b^	31.10 ± 30.19	23.90 ± 37.52	10.90 ± 35.60	0.02^f^
CRP (mg/L)	Day 0	24.00 (12.00, 48.00)	36.00 (15.00, 84.00)	48.00 (15.00, 48.00)	48.00 (24.00, 88.09)	0.31^g^
	Day 6	12.00 (6.00, 48.00)^b^	36.00 (24.00, 96.00)	48.00 (15.00, 96.00)	24.00 (12.00, 48.00)^b^ ^,^ ^d^	0.03^h^
—	Delta CRP	0.00 (−15.00, 0.00)	2.20 (0.00, 40.50)	0.00 (−24.00, 42.00)	−30.00 (−48.00, 0.00)^b^ ^,^ ^c^	0.008^h^

Data are mean ± standard deviation or median (interquartile range). LR: low risk for sepsis group, HR-C: high risk for sepsis control group, HR-DP: high risk for sepsis vitamin D and probiotics group, HR-CB: high risk for sepsis vitamin C and vitamin B1 group. MCP-1: monocyte chemoattractant protein-1, ESR: erythrocyte sedimentation rate, CRP: C-reactive protein. Delta MCP-1: change in monocyte chemoattractant protein-1 on day 6 compared to day 0, Delta ESR 1st hour: change in erythrocyte sedimentation rate value of first hour on day 6 compared to day 0, Delta ESR 2nd hour: change in erythrocyte sedimentation rate value of second hour on day 6 compared to day 0, Delta CRP: change in C-reactive protein on day 6 compared to day 0.

a = Significant with LR group.

b = Significant with HR-C group.

c = Significant with HR-DP group.

d = Significant difference between day 0 and day 6 serum level of inflammatory index within the same group. Significance level at *p*-value ≤ 0.05.

e : Analysis of variance (ANOVA) used to detect differences between groups.

f : Analysis of variance (ANOVA) followed by post-hoc test according to homogeneity of variances (Levine’s test), where we used Bonferroni post-hoc test if equal variances were assumed and Games-Howell post-hoc test if no homogeneity of variances was found. For both Bonferroni and Games-Howell post-hoc tests, the mean difference was significant at 0.05 level.

g : Kruskal–Wallis test used to detect differences between groups.

h : Kruskal–Wallis test followed by post-hoc test (Dunn’s test).

Comparing the serum MCP-1 levels within the same group, the HR-C group showed a significant increase in MCP-1 level on day 6 compared to day 0 (*p*-value = 0.014). Interestingly, both HR-DP and HR-CB groups showed a significant decrease in MCP-1 level on day 6 compared to that on day 0 (*p*-value < 0.0001).

The ESR, at the first and second hours, revealed no significant differences between groups. Within the same group, the ESR showed a significant increase in the HR-DP and HR-C groups on day 6 compared to that on day 0 at both the first and second hours (*p*-value ≤ 0.05).

The serum CRP level on day 6 revealed a significant decrease in both the LR and HR-CB groups compared to that in the HR-C group (*p*-value = 0.03). Within the same group, the HR-CB group showed a significant decrease in serum CRP level on day 6 compared to that on day 0 (*p*-value = 0.02**)**.

### 3.4 APACHE II and SOFA Scores

Monitoring the improvement (decrease) or deterioration (increase) in clinical and laboratory items of APACHE II score on day 6 compared to those on day 0 within the same group, the HR-C group showed a significant deterioration in APACHE II score (*p*-value = 0.014). Noteworthily, the LR, HR-DP, and HR-CB groups showed a significant improvement in APACHE II score on day 6 compared to their initial score on day 0 (*p*-value = 0.003, 0.003 and <0.0001 for LR, HR-DP and HR-CB groups, respectively) and a significant improvement compared to the HR-C group on day 6 (Tables 6, 7, Supplementary Figure S1A).

**TABLE 6:** Clinical and laboratory items of the Acute Physiologic Assessment and Chronic Health Evaluation score II (APACHE II) on day 0.

Variable	LR (*n* = 25)	HR-C (*n* = 20)	HR-DP (*n* = 20)	HR-CB (*n* = 20)	*p*-value
Day 0 APACHE II score	12.64 ± 4.55^a^	15.45 ± 6.57	16.30 ± 4.29	13.10 ± 3.84	0.044^c^
Variables of day 0 APACHE II score					
Temperature (°C)	36.30 (36.10, 37.00)	36.40 (36.10, 37.88)	36.55 (36.23, 37.10)	36.45 (36.20, 37.58)	0.52^d^
MAP (mmHg)	88.44 ± 10.46^a^	85.93 ± 11.48	79.59 ± 6.56	87.70 ± 11.40	0.03^c^
HR (beats/min)	101.00 (71.00,117.00)	110.00 (97.00,119.75)	108.50 (93.75,122.00)	105.00 (94.50,110.00)	0.51^d^
RR (breaths/min)	25.00 (21.50, 27.00)	22.00 (20.00, 27.50)	25.00 (21.25, 27.75)	25.00 (20.50, 27.00)	0.77^d^
GCS	13.00 (9.50, 15.00)	13.50 (10.25, 15.00)	10.00 (8.00, 14.00)	13.50 (10.00, 15.00)	0.47^d^
A-aO_2_ (mmHg)	326.90 (318.65,328.10)	322.40 (300.70,454.03)	309.60 (270.28,333.03)	325.70 (253.40,382.00)	0.86^d^
PaO_2_ (mmHg)	91.85 (69.65,124.58)	73.30 (38.88,127.5)	60.45 (40.68, 86.15)	63.90 (41.80, 82.95)	0.08^d^
Serum sodium (mmol/L)	141.00 (136.50,142.95)	142.75 (136.25,143.75)	138.00 (136.40,142.75)	140.40 (137.25,141.23)	0.28^d^
Serum potassium (mmol/L)	3.10 (2.60, 3.42)	3.33 (2.53, 3.60)	3.22 (2.70, 3.50)	3.17 (2.66, 3.40)	0.84^d^
Serum bicarbonate (mmol/L)	20.81 ± 4.16	18.95 ± 3.51	19.96 ± 3.48	19.72 ± 3.34	0.41^b^
Cr (mg/dl)	1.00 (0.82, 1.13)	1.16 (0.98, 1.50)	1.07 (1.01, 1.33)	1.05 (0.88, 1.34)	0.14^d^
HCT (%)	35.16 ± 6.32	35.45 ± 7.05	33.16 ± 6.14	34.61 ± 6.84	0.69^b^
WBCs (cells/mm^3^)	16.00 (10.25, 20.45)	17.85 (15.18, 21.40)	18.30 (14.65, 23.38)	17.40 (12.20, 21.68)	0.34^d^

Data are mean ± standard deviation, median (interquartile range). LR: low risk for sepsis group, HR-C: high risk for sepsis control group, HR-DP: high risk for sepsis vitamin D and probiotics group, HR-CB: high risk for sepsis vitamin C and vitamin B1 group, MAP: mean arterial blood pressure, HR: heart rate, RR: respiratory rate, GCS: Glasgow coma score, A-a O_2_: alveolo-arterial oxygen gradient, PaO_2_: arterial partial pressure of oxygen, HCT: hematocrit, WBC: white blood cells count.

a = Significant with HR-DP group. Significance level at *p*-value ≤ 0.05.

b : Analysis of variance (ANOVA) used to detect differences between groups.

c : Analysis of variance (ANOVA) followed by post-hoc test according to homogeneity of variances (Levine’s test), where we used Bonferroni post-hoc test if equal variances were assumed and Games-Howell post-hoc test if no homogeneity of variances was found. For both Bonferroni and Games-Howell post-hoc tests, the mean difference was significant at 0.05 level.

d : Kruskal–Wallis test used to detect differences between groups.

**TABLE 7:** Clinical and laboratory items of the Acute Physiologic Assessment and Chronic Health Evaluation score II (APACHE II) on day 6.

Variable	LR (*n* = 25)	HR-C (*n* = 20)	HR-DP (*n* = 20)	HR-CB (*n* = 20)	*p*-value
Day 6 APACHE II score	7.00 (5.50, 13.00)^a^ ^,^ ^c^	17.50 (11.75, 25.75)^c^	10.00 (6.25, 14.50)^a^ ^,^ ^c^	8.00 (5.00, 11.75) ^a^ ^,^ ^c^	<0.0001^g^
Variables of day 6 APACHE II score					
Temperature (°C)	36.40 (36.00, 37.00)^a^	35.85 (34.85, 36.33)	36.45 (36.10, 37.00)^a^	36.45 (36.00, 37.00)^a^	0.011^g^
MAP (mmHg)	89.38 ± 9.54	80.38 ± 15.93	85.65 ± 5.67	91.75 ± 7.44^a^ ^,^ ^b^	0.005^e^
HR (beats/min)	94.35 ± 21.84^a^	118.35 ± 30.77	93.90 ± 18.73^a^	89.80 ± 20.91^a^	0.001^e^
RR (breaths/min)	22.00 (20.00, 24.50)^a^	25.00 (23.50, 27.75)	22.00 (20.00, 24.00)^a^	22.00 (20.50, 25.75)^a^	0.012^g^
GCS	15.00 (13.00, 15.00)^a^ ^,^ ^b^	12.00 (5.25, 13.00)	13.00 (9.25, 15.00)	14.00 (12.00, 15.00)^a^	0.001^g^
A-aO_2_ (mmHg)	—	290.20 (265.90,324.55)	250.65 (223.20,278.1)	291.70 (231.05,332.40)	0.52^f^
PaO_2_ (mmHg)	80.80 (62.23,104.50)	72.70 (44.50, 91.10)	79.35 (65.68, 88.68)	96.70 (47.33,108.78)	0.44^f^
Serum sodium (mmol/L)	139.70 (137.00,144.05)^a^	146.00 (139.70,160.75)	140.00 (137.00,143.95)^a^	137.65 (135.00,143.00)^a^	0.006^g^
Serum potassium (mmol/L)	3.23 (2.94, 3.54)	3.20 (2.85, 3.40)	3.05 (2.83, 3.50)	3.20 (2.79, 3.58)	0.96^f^
Serum bicarbonate (mmol/L)	24.65 ± 3.86	24.12 ± 5.00	24.54 ± 4.67	24.54 ± 2.76	0.98^d^
Serum creatinine (mg/dl)	0.86 (0.70, 1.1)^a^	1.04 (0.93, 1.75)	0.83 (0.76, 1.20)	0.80 (0.67, 0.89)^a^	0.01^g^
HCT (%)	32.33 ± 6.93	32.54 ± 7.43	31.26 ± 6.40	31.58 ± 5.21	0.91^d^
WBCs (cells/mm^3^)	10.50 (7.44, 12.25)	11.65 (9.05, 13.23)	12.65 (7.90, 15.00)	10.25 (8.45, 13.18)	0.35^f^
Delta APACHE II (day 6 compared to day 0)	−3.00 (−7.00, −1.00)^a^ ^,^ ^c^	3(−0.75, 5.00)^c^	−7.00 (−8.00, −2.00)^a^ ^,^ ^c^	−4.00 (−7.50, −2.00)^a^ ^,^ ^c^	<0.0001^g^

Data are mean ± standard deviation, median (interquartile range). LR: low risk for sepsis group, HR-C: high risk for sepsis control group, HR-DP: high risk for sepsis vitamin D and probiotics group, HR-CB: high risk for sepsis vitamin C and vitamin B1 group, MAP: mean arterial blood pressure, HR: heart rate, RR: respiratory rate, GCS: Glasgow coma score, A-a O_2_: alveolo-arterial oxygen gradient, PaO_2_: arterial partial pressure of oxygen, HCT: hematocrit, WBC: white blood cells count.

a = Significant with HR-C group.

b = Significant with HR-DP group.

c = Significant difference between day 0 and day 6 score within the same group. Significance level at *p*-value ≤ 0.05. For A-aO_2_ on day 6 APACHE II, only one value existed for LR group (A-aO_2_ = 309.7 mmHg) and thus could not obtain median (IQR).

d : Analysis of variance (ANOVA) used to detect differences between groups.

e : Analysis of variance (ANOVA) followed by post-hoc test according to homogeneity of variances (Levine’s test), where we used Bonferroni post-hoc test if equal variances were assumed and Games-Howell post-hoc test if no homogeneity of variances was found. For both Bonferroni and Games-Howell post-hoc tests, the mean difference was significant at 0.05 level.

f : Kruskal–Wallis test used to detect differences between groups.

g : Kruskal–Wallis test followed by post-hoc test (Dunn’s test).

Comparing the increase or decrease in parameters of SOFA score from day 0 to day 6 within the same group, the HR-C group showed a significant deterioration in SOFA score on day 6 compared to its initial score on day 0 (*p*-value = 0.002), while the LR, HR-DP, and HR-CB groups showed a significant improvement in SOFA score (*p*-value = 0.04, 0.026, and 0.02 for LR, HR-DP, and HR-CB groups, respectively). Furthermore, a significant improvement was observed in SOFA score of the HR-DP, HR-CB, and LR groups compared to the HR-C group on day 6 (Tables 8, 9, Supplementary Figure S1B).

**TABLE 8:** Clinical and laboratory items of Sequential Organ Failure Assessment (SOFA) score on day 0.

Variable	LR (*n* = 25)	HR-C (*n* = 20)	HR-DP (*n* = 20)	HR-CB (*n* = 20)	*p*-value
Day 0 SOFA score	3.00 (2.00, 4.50)^b^	4.50 (3.00, 7.00)	6.00 (4.25, 6.75)	3.50 (3.00, 5.00)^b^	0.001^f^
Variables of SOFA score on day 0					
PaO_2_ (mmHg)	89.50 (63.75,125.15)	75.15 (56.75, 98.15)	65.30 (46.70,101.18)	66.00 (43.38, 79.1)	0.15^e^
FiO_2_	0.21 (0.21, 0.40)	0.40 (0.23, 0.60)	0.40 (0.21, 0.60)	0.21 (0.21, 0.58)	0.10^e^
PaO_2_/FiO_2_	340.00 (217.99,450.00)^a^ ^,^ ^b^	139.17 (102.53,340.50)	175.00 (133.13,242.92)	231.19 (104.00,344.29)	0.76^f^
PLT (K/uL)	166.00 (132.00,226.50)	197.00 (143.00,226.75)	146.00 (138.25,194.75)	177.00 (128.75,220.00)	0.49^e^
Bilirubin (mg/dl)	0.58 (0.49, 0.78)	0.51 (0.39, 0.77)	0.65 (0.43, 0.99)	0.67 (0.46, 1.27)	0.27^e^
MAP (mmHg)	88.44 ± 10.46^b^	85.93 ± 11.48	79.59 ± 6.56	87.7 ± 11.40	0.03^c^
On vasopressors (Dopamine, Epinephrine or Norepinephrine)	1 (4%)	2 (10%)	1 (5%)	0 (0%)	0.73^d^
GCS	13.00 (9.50, 15.00)	13.50 (10.25, 15.00)	10.00 (8.00, 14.00)	13.50 (10.00, 15.00)	0.47^e^
Serum Creatinine (mg/dl)	1.00 (0.82, 1.13)	1.16 (0.98, 1.50)	1.07 (1.01, 1.33)	1.05 (0.88, 1.34)	0.14^e^

Data are mean ± standard deviation, median (interquartile range) or number (incidence). LR: low risk for sepsis group, HR-C: high risk for sepsis control group, HR-DP: high risk for sepsis vitamin D and probiotics group, HR-CB: high risk for sepsis vitamin C and vitamin B1 group. PaO_2_: arterial partial pressure of oxygen, FiO_2_: fraction of inspired oxygen, PLT: platelets, MAP: mean arterial blood pressure, GCS: Glasgow coma score.

a = Significant with HR-C group.

b = Significant with HR-DP group. Significance level at *p*-value ≤ 0.05.

c : Analysis of variance (ANOVA) followed by post-hoc test according to homogeneity of variances (Levine’s test), where we used Bonferroni post-hoc test if equal variances were assumed and Games-Howell post-hoc test if no homogeneity of variances was found. For both Bonferroni and Games-Howell post-hoc tests, the mean difference was significant at 0.05 level.

d : Monte Carlo test with 95% confidence interval used to detect differences among groups.

e : Kruskal–Wallis test used to detect differences among groups.

f : Kruskal–Wallis test followed by post-hoc test (Dunn’s test).

**TABLE 9:** Clinical and laboratory items of Sequential Organ Failure Assessment (SOFA) score on day 6.

Variable	LR (*n* = 25)	HR-C (*n* = 20)	HR-DP (*n* = 20)	HR-CB (*n* = 20)	*p*-value
Day 6 SOFA score	2.00 (1.00, 3.50)^a^ ^,^ ^b^ ^,^ ^c^	7.00 (3.00, 9.75)^c^	4.00 (2.25, 6.00) ^c^	2.00 (1.00, 4.00)^a^ ^,^ ^b^ ^,^ ^c^	<0.0001^g^
Variables of SOFA score on day 6					
PaO_2_ (mmHg)	79.80 (60.85,100.45)	78.05 (42.78, 94.20)	80.85 (67.10, 99.40)	95.95 (51.60, 109.45)	0.48^f^
FiO_2_	0.21 (0.21, 0.22)^a^	0.51 (0.40, 0.60)	0.21 (0.21, 0.40)^a^	0.21 (0.21, 0.40)^a^	<0.0001^g^
PaO_2_/FiO_2_	353.81 (221.67,460.71)^a^	157.08 (88.46, 227.60)	254.29 (181.55, 391.18)^a^	284.25 (184.12, 464.64)^a^	0.001^g^
PLT (K/uL)	174.00 (147.50, 228.50)^b^	166.00 (105.75, 249.00)	127.50 (108.75, 181.00)	180.00 (165.50, 232.25) ^b^	0.04^g^
Bilirubin (mg/dl)	0.69 (0.52, 0.96)	0.81 (0.56, 1.10)	0.80 (0.63, 1.15)	0.60 (0.45, 0.93)	0.38^f^
MAP (mmHg)	89.38 ± 9.54	80.38 ± 15.93	85.65 ± 5.67	91.75 ± 7.44^a^ ^,^ ^b^	0.005^d^
On Vasopressors (Dopamine, Epinephrine or Norepinephrine)	0 (0%)^a^	7 (35%)	0 (0%)^a^	0 (0%)^a^	<0.0001^e^
GCS	15.00 (13.00, 15.00)^a^ ^,^ ^b^	12.00 (5.25, 13.00)	13.00 (9.25, 15.00)	14.00 (12.00, 15.00)^a^	0.001^g^
Serum Creatinine (mg/dl)	0.86 (0.70, 1.10)^a^	1.04 (0.93, 1.75)	0.83 (0.76, 1.20)	0.80 (0.67, 0.89)^a^	0.01^g^
Delta SOFA (on day 6 compared to day 0)	−1.00 (−2.00, 0.50)^a^ ^,^ ^c^	2.00 (0.00, 4.50)^c^	−2.00 (−3.00, 0.75)^a^ ^,^ ^c^	−1.50 (−3.00, 0.00)^a^ ^,^ ^c^	<0.0001^g^

Data are mean ± standard deviation, median (interquartile range) or number (incidence). LR: low risk for sepsis group, HR-C: high risk for sepsis control group, HR-DP: high risk for sepsis vitamin D and probiotics group, HR-CB: high risk for sepsis vitamin C and vitamin B1 group. PaO_2_: arterial partial pressure of oxygen, FiO_2_: fraction of inspired oxygen, PLT: platelets, MAP: mean arterial blood pressure, GCS: Glasgow coma score.

a = Significant with HR-C group

b = significant with HR-DP group.

c = Significant difference between day 0 and day 6 score within the same group. Significance level at *p*-value ≤ 0.05.

d : Analysis of variance (ANOVA) followed by post-hoc test according to homogeneity of variances (Levine’s test), where we used Bonferroni post-hoc test if equal variances were assumed and Games-Howell post-hoc test if no homogeneity of variances was found. For both Bonferroni and Games-Howell post-hoc tests, the mean difference was significant at 0.05 level.

e : Monte Carlo test with 95% confidence interval followed by post-hoc test where significant *p*-value is determined against adjusted *α* = 0.00625 (when using Bonferroni correction).

f : Kruskal Wallis test used to detect differences among groups.

g : Kruskal Wallis test followed by post-hoc test (Dunn’s test).

### 3.5 Patients Who Completed Treatment Regimen in the ICU, but Transferred Outside, Died, or Discharged to Ward Before Day 6

Three, three, two, and one patient in the LR, HR-C, HR-DP, and HR-CB groups, respectively, died or were discharged home before day 6 but after completing the study treatment regimen in the ICU. However, two, one, two, and four patients in the LR, HR-C, HR-DP, and HR-CB groups, respectively, were discharged to the ward before day 6 and after completion of the study regimen in the ICU.

### 3.6 Sepsis Development

**FIGURE 2:**
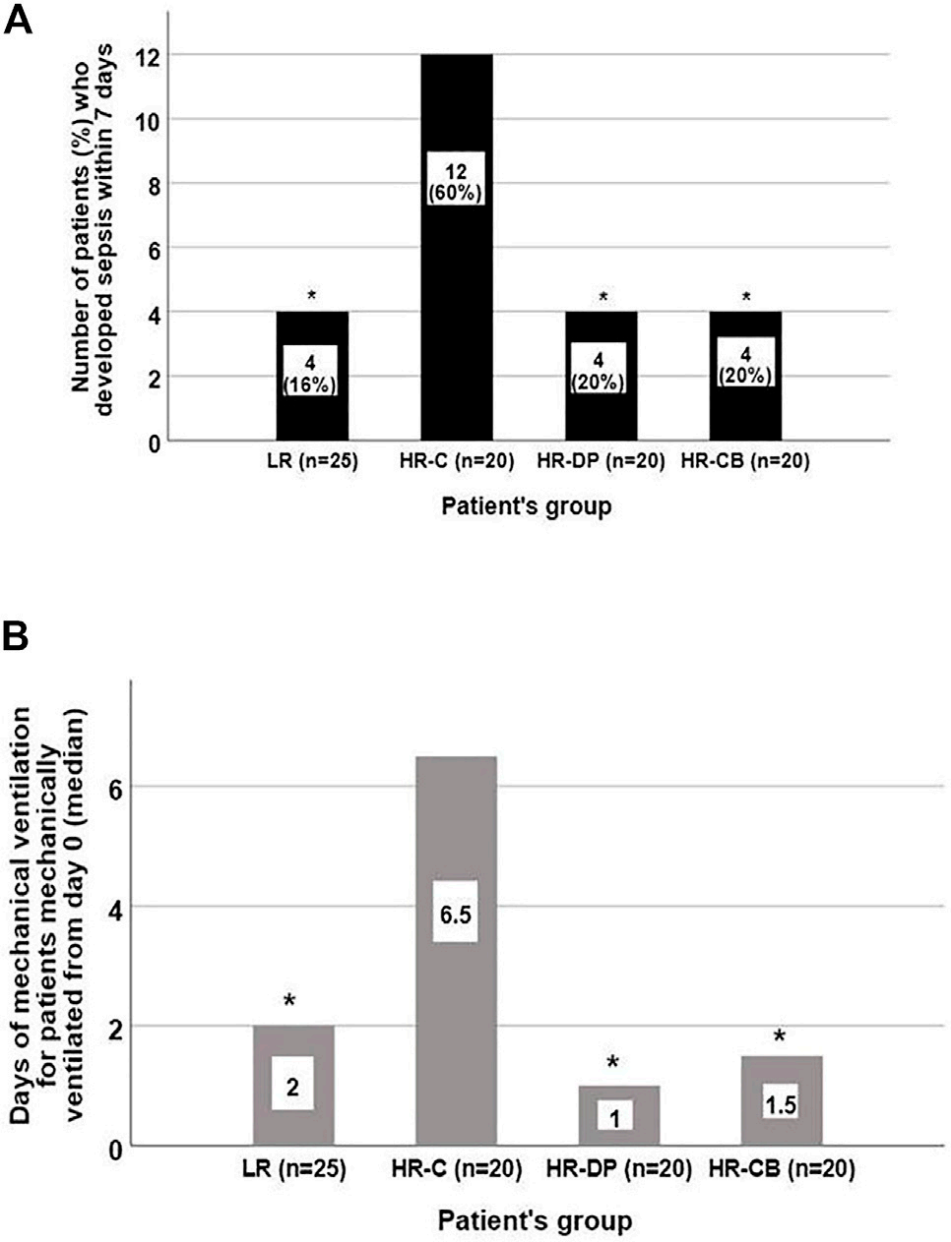
Number of patients who developed sepsis besides the duration of mechanical ventilation for patients mechanically ventilated from day 0 in each group by the end of the first week. **(A)** Number of patients developing sepsis (by the end of the first week) in each group. **(B)** Days of mechanical ventilation for patients ventilated from day 0 in each group. (* = significant compared to HR-C group, *p*-value ≤ 0.05). LR: low risk for sepsis group, HR-C: high risk for sepsis control group, HR-DP: high risk for sepsis vitamin D and probiotics group, HR-CB: high risk for sepsis vitamin C and vitamin B1 group. Data are number (incidence), median (IQR).

The incidence of sepsis by the end of the first week in each group according to the sepsis-3 criteria (Singer et al., 2016) is presented in Figure 2A. The highest incidence of sepsis development was revealed in the HR-C group compared to the other three groups (*p*-value = 0.004). The coagulase negative *Staphylococcus aureus* (CONS) represented the most abundant species isolated from positive aerobic blood cultures in all groups (Supplementary Figure S2).

### 3.7 Duration of Mechanical Ventilation

Patients in the LR, HR-DP, and HR-CB groups who needed mechanical ventilation upon admission had a significantly shorter duration of mechanical ventilation compared to the HR-C group by the end of the first week (*p*-value = 0.014) (Figure 2B).

### 3.8 Mortality, ICU Discharge, and Hospital Discharge

**FIGURE 3:**
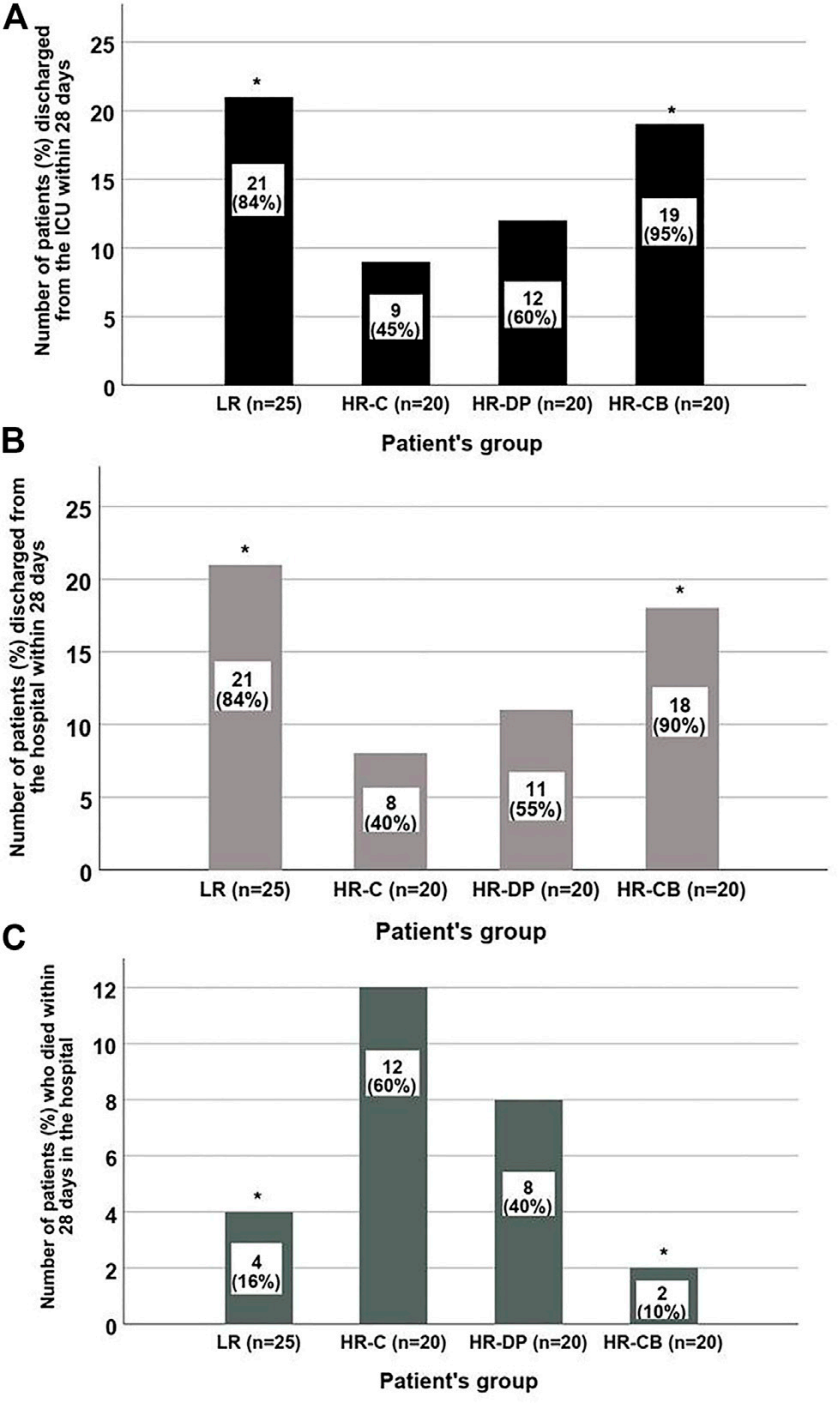
Number of patients discharged from the ICU, from the hospital or died during the first 28 days in each group. **(A)** ICU discharge within 28 days. **(B)** Hospital discharge within 28 days. **(C)** The 28-day hospital mortality in each group (* = significant compared to HR-C group, *p*-value ≤ 0.05). LR: low risk for sepsis group, HR-C: high risk for sepsis control group, HR-DP: high risk for sepsis vitamin D and probiotics group, HR-CB: high risk for sepsis vitamin C and vitamin B1 group. Data are number (incidence).

During the first 28 days, both LR and HR-CB groups showed a significant increase in ICU (*p*-value = 0.001) and hospital discharge (*p*-value = 0.001) (Figures 3A,B) in addition to a significant decrease in mortality incidence (*p*-value = 0.001) (Figure 3C) compared to the HR-C group.

### 3.9 Incidence of AKI

To evaluate the occurrence of AKI among the study population, serum creatinine levels on day 6 were compared to the initial values on day 0. It was observed that 10 patients developed AKI. These patients were distributed as follows: two, five, one, and two patients in the LR, HR-C, HR-CB, and HR-DP groups, respectively, with no statistically significant differences (*p*-value = 0.22).

### 3.10 Survival Analysis and Multivariate Cox Proportional Hazard Model

**FIGURE 4:**
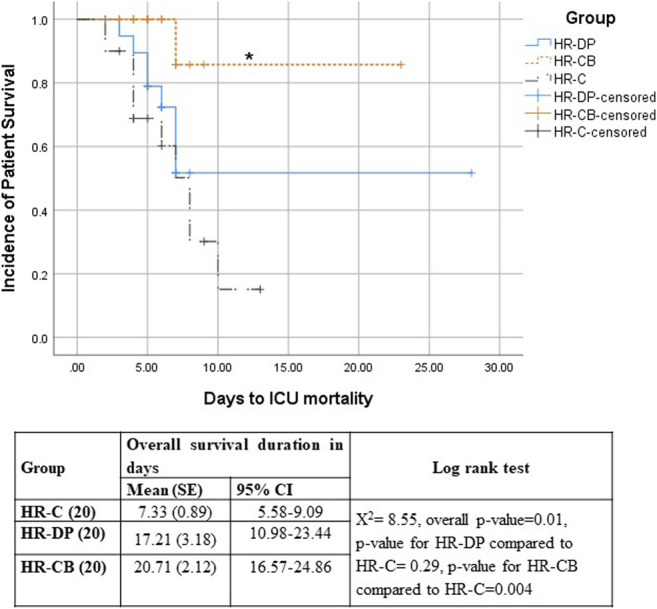
Kaplan–Meier survival estimates of ICU Mortality HR-C: High risk for sepsis control group, HR-DP: High risk for sepsis vitamin D and probiotics group, HR-CB: High risk for sepsis vitamin C and vitamin B1 group, SE: standard error, 95% CI: 95% confidence interval, *ꭕ* ^2^: chi-square, * = Significant compared to HR-C group, *p*-value ≤ 0.05.

Survival analysis showed that the HR-CB group had a significantly lower ICU mortality compared to the HR-C group (Figure 4). The univariate Cox proportional hazard models showed significance for both the effect of study treatment (*p*-value = 0.022 and 0.309 for HR-CB and HR-DP, respectively, compared to HR-C group) and sepsis development (*p*-value = 0.009), whereas all the other tested variables in HR groups were non-significant (*p*-value > 0.05). Hence, the multivariate Cox proportional hazard model was performed using effect of study treatment and sepsis development as covariates (Table 10). Patients who developed sepsis by the end of the first week had a significantly higher hazard of ICU mortality than those who did not develop sepsis (hazard ratio = 3.291; *p* = 0.034; 95% CI, 1.097–9.869). Regarding the effect of study treatment versus control in HR groups, the HR-CB group showed the lowest hazard ratio for ICU mortality compared to the HR-C group. However, the difference between hazard ratios did not reach the threshold of statistical significance (hazard ratio = 0.137; *p* = 0.06; 95% CI, 0.017–1.091).

**TABLE 10:** Multivariate Cox regression model of risk factors for ICU mortality during the first 28 days from the onset of trauma.

	*p*-value	Hazard ratio	95% Confidence interval	
			Lower	Upper
HR-C (reference group)	0.137	—	—	—
HR-DP group	0.833	1.130	0.363	3.516
HR-CB group	0.060	0.137	0.017	1.091
Sepsis development	0.034^a^	3.291	1.097	9.869

HR-C: high risk for sepsis control group, HR-DP: high risk for sepsis vitamin D and probiotics group, HR-CB: high risk for sepsis vitamin C and vitamin B1 group.

a = Significant compared to no sepsis development by the end of the first week. Significance level at *p*-value ≤ 0.05.

### 3.11 Evaluating Predictive Value of Different Sepsis Predictors in the No-Intervention Groups

**FIGURE 5:**
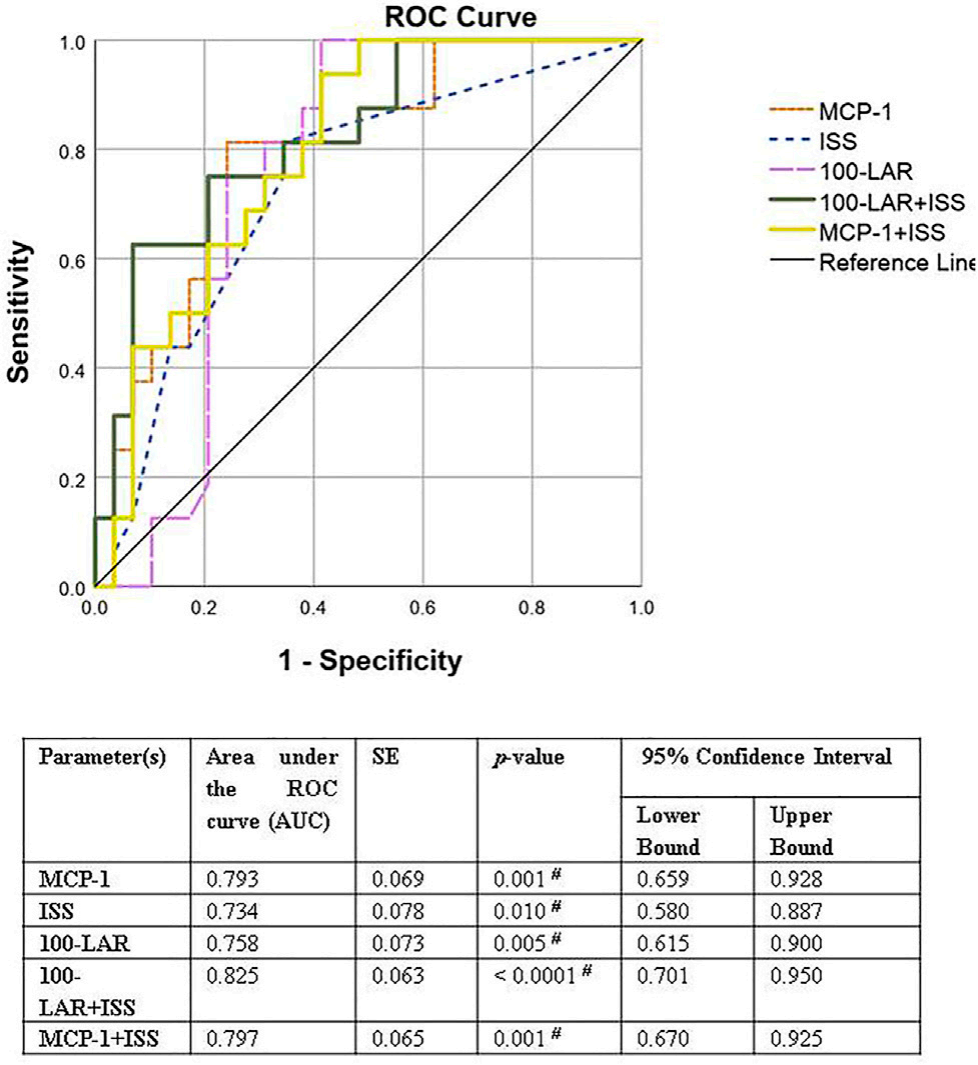
Receiver operating characteristics (ROC) curve for predictive value of different sepsis predictors among non-intervention groups (HR-C and LR groups). MCP-1: Monocyte chemoattractant protein-1, ISS: Injury severity score. LAR: Leukocyte anti sedimentation rate. SE: standard error. # = Significant *p*-value. Significance level at *p* ≤ 0.05.

The predictive value for different sepsis predictors was evaluated in the no-intervention groups (HR-C and LR). Areas under the ROC curve (AUCs) of MCP-1 (day 0), ISS (day 0), and 100-LAR (day 1) were 0.793 (95% CI, 0.66–0.93; *p*-value = 0.001), 0.734 (95% CI, 0.58–0.89; *p*-value = 0.01), and 0.758 (95% CI, 0.62–0.9; *p*-value = 0.005), respectively (Figure 5). Hence, the test performance of each predictor alone was fair (Hosmer et al., 2013). Combining the predictors, MCP-1 + ISS and 100-LAR + ISS, yielded higher AUCs of 0.797 and 0.825, respectively. Therefore, the combined use of either MCP-1 or LAR with ISS was better than each indicator alone. The test performance for combined predictors was good (Hosmer et al., 2013) with higher sensitivity for MCP-1 + ISS compared to higher specificity for 100-LAR + ISS. Sensitivity and specificity for MCP-1 + ISS were 94% and 59%, respectively. Conversely, sensitivity and specificity for 100-LAR + ISS were 63% and 93%, respectively. Optimal cutoff values to predict sepsis were determined on the ROC curve with maximum Youden-index [sensitivity − (1 − specificity)]. The best thresholds of MCP-1, 100-LAR, and ISS for sepsis prediction were 138.98 pg/ml, 70.85%, and 16.5, respectively.

### 3.12 Safety and Adverse Effects

Throughout the patients’ follow-up, few complications were recorded. Two patients in the HR-CB group showed hypersensitivity (positive IDT for vitamin B1) with no other complications. Consequently, these patients were excluded from the study. No other adverse events were deemed related to the study drugs in the HR-CB and HR-DP groups in the entire study period.

## 4 Discussion

### 4.1 The Effect of Study Drugs on Patient Clinical Outcomes

In the current study, LAR was used for determination of patients who have high risk for sepsis development. The effects of immunomodulatory interventions (IV vitamin C plus vitamin B1 versus IM vitamin D plus oral probiotics) on prevention of sepsis development were investigated among patients with major trauma at high risk for sepsis. Both interventions decreased the incidence of sepsis development to the same extent (20%). However, vitamin C plus vitamin B1 were associated with lower 28-day mortality rate and higher ICU and hospital discharge rates than vitamin D plus probiotics.

### 4.2 The Proposed Mechanisms for Vitamin D Plus Probiotics’ Effects on Sepsis and Inflammation

This current study showed that vitamin D plus probiotics significantly decreased scores for illness severity (APACHE II and SOFA), proinflammatory biomarker MCP-1, and sepsis development. The overall good clinical outcomes observed in the HR-DP group may be attributed to the synergistic effects of vitamin D plus probiotic combination. The benefits of vitamin D and probiotic cosupplementation on inflammation and antioxidant capacity have been studied in other contexts than ICU severe trauma (Abboud et al., 2021). *Lactobacillus fermentum*, one of the components of probiotic product used in this study, is among the most studied *Lactobacilli* strains with antimicrobial activity (de Melo Pereira et al., 2018; Silva et al., 2020). The antimicrobial effect of probiotics may be attributed to their gut barrier protective effects (Crooks et al., 2012; Assimakopoulos et al., 2018). Probiotics block adhesion sites of pathogenic microorganisms in the intestinal mucosa, compete with them for nutrients, and produce antibacterial substances during their elimination process. These substances include lactic acid, bacteriocin, exopolysaccharides, and hydrogen peroxide (Bermudez-Brito et al., 2012). Bacteriocin has been used by researchers to synthesize probiotic-derived bacteriocin-modified antimicrobial peptides. These peptides demonstrated strong antibacterial activity against multidrug-resistant bacteria in preclinical studies and are expected to replace antibiotics in the future (Mazumdar et al., 2020). Besides, vitamin D supplementation has been suggested for sepsis prevention in the critically ill due to its immunomodulatory effects (Takeuti et al., 2018). The anti-inflammatory characteristics of probiotics are dependent on vitamin D receptor (VDR) expression, and alternatively, probiotics in preclinical studies enhanced VDR and m-RNA antimicrobial cathelicidin expression (Yoon and Sun, 2011). Both high-dose vitamin D and probiotics have been studied separately among the ICU trauma population and showed potential benefits (Kotzampassi et al., 2006; Hasanloei et al., 2020).

### 4.3 The Possible Mechanisms of Vitamin C Plus Vitamin B1’s Influence on Sepsis Prevention

The overall improved patient outcomes in the HR-CB group compared to the HR-C group could be attributed to the synergistic effect of vitamin C plus vitamin B1, which could be explained by a twofold mechanism. First, both vitamin C and vitamin B1 have an anti-inflammatory effect *via* inhibition of nuclear factor kappa B signaling, antioxidant potential, and mitochondrial protective mechanisms (Marik, 2018). The effects of vitamin C and vitamin B1 on mitochondrial biogenesis are critical elements in their sepsis-preventing effects compared to N-acetyl cysteine, whose unproven effects were attributed to its low ability to enter the mitochondria (Molnár, 2008). Second, vitamin B1 mitigates vitamin C-induced renal toxicity by acting as a cofactor for glyoxylate aminotransferase, the enzyme that converts glyoxylate (metabolic product of vitamin C) to carbon dioxide instead of oxalate, which causes nephropathy (Oudemans-van Straaten et al., 2017). Besides that, vitamin C supplementation enhances both innate and adaptive immunity (Carr and Maggini, 2017). The antibacterial effect of vitamin C is both concentration and bacterial strain dependent (Kallio et al., 2012; Mehmeti et al., 2013). Vitamin C has been shown to act synergistically with some antibiotics against different types of bacteria in previous studies such as synergism with rifampicin and isoniazid against multidrug-resistant *Staphylococcus aureus* and *Mycobacterium tuberculosis* isolates (Khameneh et al., 2016; Pandit et al., 2017). Vitamin C has also been suggested as an antibiotic modifier acting synergistically with chloramphenicol, kanamycin, streptomycin, and tetracycline against multi-resistant *Pseudomonas aeruginosa* isolates obtained from burn patients (Cursino et al., 2005). Additionally, both trauma and sepsis fall under the umbrella of endothelial dysfunction-dependent pathophysiology (Lehr et al., 2006). Vitamin C reduces endothelial dysfunction and capillary leakage syndrome by reducing detachment in tight gap junctions, detoxification of histamine, and synthesis of endogenous vasopressors (Carr et al., 2015).

### 4.4 The Predictive Value of the LAR Test

In this study, the fair test performance of LAR (AUC, 0.758) as a predictor of sepsis is concordant with a previous study reporting good test performance of LAR (AUC, 0.8) as a predictor of bacteremia in a general surgical ICU population (Bogar et al., 2006). Furthermore, combining LAR with ISS further increased AUC to 0.825, resulting in a good test performance comparable to that of MCP-1 and ISS (AUC of 0.87) reported in a previous study (Wang et al., 2018).

### 4.5 Similar Previous Studies in Trauma Patients

The significant decrease in SOFA score and consequently the significantly lower incidence of sepsis among the intervention groups compared to control were concordant with previous studies conducted on the use of synbiotics (Kotzampassi et al., 2006), vitamin D (Hasanloei et al., 2020), vitamin C, and N-acetyl cysteine (Sandesc et al., 2018) among the ICU trauma population. However, in a previous trial using 300,000 IU vitamin D in trauma, patients did not develop sepsis (Hasanloei et al., 2020). Compared to this study; the difference in sepsis development may be attributed to the many variable comorbidities in this study that were not mentioned in the study by Hasanloei et al. (2020). Patient comorbidities have been shown to be risk factors for sepsis development in other previous literature (Kisat et al., 2013).

Bedreag et al. found no reduced incidence of sepsis development with the use of vitamin C, vitamin B1, and N-acetyl cysteine together among ICU trauma patients (Bedreag et al., 2015). However, no exclusion criteria were stated in their retrospective study. As known, patients with immune suppression (iatrogenic or caused by a disease) are much more vulnerable to sepsis development (Kumar et al., 2015). Thus, they were excluded from our study. Moreover, Wiley et al., after administration of vitamin C and vitamin B1 in trauma, found a significantly lower peak SOFA score in the intervention group compared to that in the control group on day 3 (a concordant finding with this study’s results). However, they recorded no significant effect on shock resolution (Tessa et al., 2018).

### 4.6 The Rationale for Timing of Collection of Reserve Samples

If any patient was discharged to the ward before day 6 after completing the study treatment regimen in the ICU, final SOFA score and blood culture were collected in the ward on day 6. However, the last APACHE II score in the ICU just before discharge was recorded and forwarded for assessment. This is based on evidence from literature that full SOFA score is the best tool for identifying patients with sepsis in the ward setting (better than quick SOFA) (Szakmany et al., 2018). However, the APACHE II score represents a physiologically based ICU scoring system for measuring illness severity. The APACHE II was used to predict in-hospital mortality (incorporated both death in the ICU and the ward) for critical care patients (Knaus et al., 1981; Cardoso and Chiavone, 2013). The evidence from literature showed that discharge APACHE II score (calculated 24 h prior to ICU discharge) was related to mortality after ICU discharge. The discharge APACHE II scores of ≥17 were associated with poor post-ICU prognosis (Cardoso and Chiavone, 2013).

### 4.7 Sepsis and Mortality

All three groups (LR, HR-CB, and HR-DP) revealed a significantly lower incidence of sepsis than the HR-C group by the end of the first week. Consequently, these three groups showed lower 28-day mortality than the HR-C group. Multivariate Cox regression showed that sepsis development was a significant risk factor for ICU mortality in HR groups. These results comply with a previous study reporting sepsis as a leading cause of mortality contributing to 11 million deaths annually worldwide (Rudd et al., 2020). Another study conducted in all trauma centers of Pennsylvania also showed that sepsis was associated with significantly higher mortality in patients with trauma (Osborn et al., 2004).

### 4.8 The MCP-1 in the Current and Previous Studies

Differently from this study, previous studies investigating the effects of interventions (CB and DP) on MCP-1 levels were either preclinical or clinical on patients without trauma (Dong et al., 2011; Alvarez et al., 2013; Lauer et al., 2021). The significantly reduced incidence of sepsis development by the end of the first week in both the HR-DP and HR-CB groups compared to the HR-C group was accompanied by a significant reduction in the proinflammatory chemokine MCP-1 level within these intervention groups compared to a significant increase within the control group. These results comply with a previous study revealing the key role of MCP-1 in sepsis pathogenesis (Zhu et al., 2017). Moreover, these results confirm Wang et al.’s hypothesis (Wang et al., 2018) that lowering MCP-1 level might confer an associated clinical progress in ICU patients with major trauma as decreasing MCP-1 level was accompanied by a significant reduction in incidence of sepsis development. The significant reduction in proinflammatory chemokine MCP-1 level on day 6 was concordant with previous studies on patients with trauma, but these studies investigated IL-6 as a proinflammatory cytokine (Kotzampassi et al., 2006; Sandesc et al., 2018; Hasanloei et al., 2020). Furthermore, preclinical studies suggest that the nephroprotective effects of vitamin D involve MCP-1 lowering mechanisms (Arfian et al., 2020). This was manifested by the significantly decreased MCP-1 level within the HR-DP group accompanying the reduced incidence of AKI in the HR-DP group compared to that in the HR-C group.

Alvarez et al. have shown that vitamin D inhibited MCP-1 production in patients with early CKD and *in vitro* study. The 1,25 dihydroxyvitamin D concentration used in Alvarez et al. ’s *in vitro* study (16 ng/ml) was in the range of 25-hyroxivitamin D levels of patients in the HR-DP group (10–30 ng/ml) (Alvarez et al., 2013). The effect of probiotics as MCP-1 inhibitors has been shown previously in preclinical studies concordant with this study’s findings (Dong et al., 2011; Wachi et al., 2014). In the Dong et al.’s study conducted on many Lactobacilli strains, MCP-1 levels were lower than those in positive controls (Dong et al., 2011). Another study declared that exopolysaccharides of *Lactobacillus delbrueckii* TUA4408L act on intestinal epithelial cells via toll-like receptors 2 and 4, leading to decreased production of MCP-1 (Wachi et al., 2014). *Lactobacillus delbrueckii* is one of the two probiotic strain constituents of the probiotic product used in this study (Lacteol Forte [package insert], 2018). The results of the *ex vivo* study of Lauer et al. (2021) support the findings of the significantly reduced MCP-1 level on day 6 within the HR-CB group. The average steady-state serum vitamin C concentration in the HR-CB group [0.4 mM, estimated based on dosing rate (1 g every 12 h), salt value (0.889), and clearance (0.92 L/h)] is within the range of vitamin C concentration as investigated in the Lauer et al. study (0.2–2 mM) (Lauer et al., 2021).

### 4.9 The ESR and CRP Changes in This Study Compared to Similar Previous Studies

A significant increase in ESR and a nonsignificant increase in CRP level were found within both HR-DP and HR-C groups on day 6 compared to those on day 0. However, in the HR-CB group, a nonsignificant increase in ESR besides a significant reduction in CRP level were observed on day 6 compared to those on day 0. These ESR and CRP changes in HR groups agreed with previous studies reporting the higher sensitivity of CRP to changes in acute phase response than ESR (Markanday, 2015). Kotzampassi et al. (2006) reported a significantly lower CRP level in the synbiotics group with respect to placebo on day 7. However, within the synbiotics group, no significant decrease in CRP level on day 7 compared to that on day 0 was reported. Perhaps, probiotics could not significantly lower CRP level within the HR-DP group similar to Kotzampassi et al. who used a larger dose of synbiotics (Kotzampassi et al., 2006).

#### 4.9.1 The Proposed Explanation for CRP Changes in HR-DP and HR-CB Groups

Controversially, Hasanloei et al. (2020) found a significant reduction in ESR and CRP levels in the IM vitamin D group on day 7 compared to those at baseline. One explanation for the nonsignificantly different CRP level on day 6 compared to that on day 0 in the HR-DP group could be the inverse relationship between CRP and vitamin D levels reported in literature that occurs only at serum vitamin D levels <53 nmol/L (21.2 ng/ml, conversion factor 2.496) (Cannell et al., 2014). As patients in the HR-DP group received vitamin D plus probiotics on day 1, the IM 400,000 IU vitamin D dose was expected to increase serum vitamin D level by 25 ng/ml according to Amrein et al. (2011) to reach the level of approximately 41 ng/ml on day 3 and remain on that level for 1 month (Amrein et al., 2014). Therefore, the vitamin D level on day 6 was probably ≥21.2 ng/ml in most patients in the HR-DP group; thus, the inverse relationship between vitamin D and CRP levels was no longer obvious. The IM vitamin D dose in this study exceeded that of Hasanloei et al. by 100,000 IU (Hasanloei et al., 2020). Hence, there were probably more patients with vitamin D levels ≥21.2 ng/ml in the HR-DP group than in the IM vitamin D group of Hasanloei et al. (2020).

The CRP level of the HR-CB group on day 6 was significantly lower compared to that in the control group. This result was concordant with the previous study of Sandesc et al. who found a significant decrease in CRP level and ESR in the intervention group compared to those in the control group upon ICU discharge (Sandesc et al., 2018).

#### 4.9.2 The Suggested Explanation for the ESR Changes in HR-DP and HR-CB Groups

The ESR showed an increase that was significant within the HR-DP group and insignificant within the HR-CB group on day 6 compared to that on day 0. One explanation is that fibrinogen and immunoglobulin G are the main proteins influencing ESR. Both fibrinogen and immunoglobulin G have long half-lives (Litao and Kamat, 2014). Thus, elevated ESR can take weeks to return to normal and can stay elevated after inflammation has resolved (Litao and Kamat, 2014). Perhaps in this study, if ESR had been measured after 2 weeks, it might have decreased in the HR-DP and HR-CB groups as mentioned in the study by Sandesc et al. in which ESR significantly decreased after approximately 14 days in the vitamin C and N-acetyl cysteine group compared to the control group (Sandesc et al., 2018).

### 4.10 The Rationale for Using Aerobic Bacterial Blood Cultures

Aerobic bacterial blood cultures were used as a possible documentation for infection due to their prevalence in sepsis diagnosis among the critically ill rather than anaerobic bacteria, fungi, or viruses (Dolin et al., 2019; Gajdács and Urbán, 2020). The most prevalent bacterial strain detected in aerobic bacterial positive blood cultures was CONS concordant with previous studies in Egypt (Ahmed et al., 2009) and the United States (Edmond et al., 1999).

### 4.11 The Duration of Mechanical Ventilation in this Trial Compared to a Previous Similar Trial

Patients in the HR-CB group mechanically ventilated from day 0 showed a significantly shorter duration of mechanical ventilation compared to those in the HR-C group by the end of the first week. These findings were discordant with the results of Sandesc et al., who attributed the nonsignificant difference in duration of mechanical ventilation between their groups to the high prevalence of thoracic trauma and pulmonary infections (Sandesc et al., 2018). However, in the current study, multiple trauma was the most common, followed by head trauma. The highest percentage of thoracic trauma in this study was 10% in the HR-C group, which showed the longest duration of mechanical ventilation supporting the hypothesis of Sandesc et al. (2018). The reduced duration of mechanical ventilation in the HR-CB group may be attributed to the antioxidant effects of vitamin C plus vitamin B1, which agrees with a previous meta-analysis conducted on this subject (Hemilä and Chalker, 2019).

### 4.12 The Protective Effects of Vitamin B1 and Vitamin D Against AKI

At the end of the first week, the occurrence of AKI detected by comparing day 6 and day 0 serum creatinine values followed the KDIGO guidelines. The KDIGO guidelines define the AKI as an increase in serum creatinine level to 1.5 times the baseline creatinine or more within the last 7 days (Khwaja, 2012). AKI is reported in literature as a complication of oxalate nephropathy (secondary to high dose IV vitamin C) and hypercalcemia (secondary to hypervitaminosis D) (Lamarche et al., 2011; Graidis et al., 2020). However, by monitoring the reported adverse effects, AKI was the least common in the HR-CB group [1 (5%)] and the most common in the HR-C group [5 (25%)], which could be explained by the addition of IV vitamin B1 in the HR-CB group with its renoprotective effects (Moskowitz et al., 2017). Vitamin B1 mitigates oxalate nephropathy, a side effect reported with high-dose vitamin C (Hoppe et al., 2009). Additionally, the HR-DP group also showed a lower incidence of AKI [2 (10%)] compared to the HR-C group [5 (25%)]. Vitamin D deficiency (<15 ng/ml) or insufficiency (15–30 ng/ml) predicts increased risk of AKI development (Braun et al., 2012). All patients in the HR-DP and HR-C groups had basal vitamin D levels of 10–30 ng/ml and consequently had an increased risk for AKI development. After supplementation with 400,000 IU vitamin D in the HR-DP group, serum 25-hydroxyvitamin D level was expected to reach the level of 41 ng/ml (bypassed the range associated with increased risk of AKI) (Amrein et al., 2011). The incidence of AKI in the HR-DP group was lower than that in the HR-C group, confirming the nephroprotective effects of vitamin D supplementation.

### 4.13 Strengths of the Study

One strength for this study is that, to the best of our knowledge, it is the first study to demonstrate the lowering effects of vitamin C plus vitamin B1 (CB) and vitamin D plus probiotic (DP) combinations on MCP-1 in ICU trauma patients. The average estimated serum vitamin C level in this trial (0.4 mM) was far from the level reported in a previous preclinical study to be associated with prooxidant effects (2 mM) (Park and Lee, 2008). Besides, the parenteral route of vitamin C administration bypassed the vitamin C intestinal uptake ceiling effect that occurs with oral route and is responsible for its inefficacy in critically ill patients (van Zanten et al., 2014). The use of continuous infusion rather than bolus injection fostered lower excretion of vitamin C and oxalate (de Grooth et al., 2018). The use of single IM vitamin D dose avoided the problems of slow absorption and low bioavailability encountered with oral doses (Hasanloei et al., 2020). Moreover, the use of LAR enabled sepsis-risk prediction that was not possible with leukocyte count due to its limited prognostic value (Hesselink et al., 2020). The LAR can be used as an affordable and easy method for sepsis prediction until newer methods for assessment of neutrophil dysfunction become available (Hesselink et al., 2019). The LAR combined with ISS were good sepsis predictors comparable to MCP-1 combined with ISS suggested by Wang et al. (2018), with a much lower cost.

### 4.14 Limitations of the Study

This study may be limited by the inability to measure vitamin C and vitamin B1 levels at baseline due to the requirement of high-performance liquid chromatography (HPLC), which was expensive and unavailable. Moreover, HPLC may be unable to detect the very low levels of vitamin C in critical illness (Long et al., 2003; Collie et al., 2017). Vitamin D level after supplementation could not be measured due to financial limitations. However, it was expected to be normalized and exceed the level found in a similar study of Hasanloei et al. on 300,000 IU of IM vitamin D [where mean ± SD serum 25-hyroxyvitamin D level in the IM vitamin D group on day 7 was 29.43 ± 5.18 ng/ml (Hasanloei et al., 2020)] due to the higher IM vitamin D dose in this study (400,000 IU). The probability of patient transfer outside hospital or death after completing the supplementation regimen in the ICU and before day 6 prompted the investigators of this study to collect a reserve sample and blood culture on day 3, which was used in case day 6 sample and blood culture could not be collected.

## 5 Conclusion

Early prediction of sepsis in severe trauma represents an unmet clinical need. The use of LAR as a point-of-care test combined with ISS as a cheap and available alternative to MCP-1 plus ISS enabled determination of patients at high risk for sepsis development who would benefit most from the prophylactic immunomodulatory interventions. Vitamin D plus probiotics synergistic combination reduced the incidence of sepsis development similar to IV vitamin C plus vitamin B1 in the ICU patients with trauma. Both combinations reduced MCP-1 level, proving the therapeutic progress accompanying MCP-1 level decrease in severe trauma. Hence, the administration of immunomodulatory interventions for prevention of sepsis in clinical practice could help improve major trauma patient prognosis and decrease the incidence of sepsis.

## Data Availability

The raw data supporting the conclusion of this article will be made available by the authors, upon request.

## Supplementary Material

Supplementary Figure S1 APACHE II and SOFA scores on day 0 and day 6 in the tested groups, **(A)**: APACHE II score on day 0 and day 6, **(B)**: SOFA score on day 0 and day 6.

Supplementary Figure S2 The number of patients (incidence) with positive aerobic bacterial blood culture (obtained on day 6) in each group and the incidence of bacterial strains in each group.

## Abbreviations

APACHE II, acute Physiologic Assessment and Chronic Health Evaluation score II; CB, vitamin C plus vitamin B1; CRP, C-reactive protein; DP, vitamin D plus probiotics; ELISA, enzyme-linked immunosorbent assay; ESR, erythrocyte sedimentation rate; HR, high-risk; HR-DP, high-risk vitamin D plus probiotics; HR-CB, high-risk vitamin C plus vitamin B1; IL-6, interleukin-6; IDT, intradermal skin test; ISS, injury severity score; LAR, leukocyte anti-sedimentation rate; LR, low-risk; MCP-1, monocyte chemoattractant protein-1; ROC curve, receiver operating characteristics curve; SOFA, sequential Organ Failure Assessment.
