# Supplementary material for: Effect of Anti-Inflammatory and Antimicrobial Cosupplementations on Sepsis Prevention in Critically Ill Trauma Patients at High Risk for Sepsis
Source: Front Pharmacol. 2021 Nov 29;12:792741. doi: 10.3389/fphar.2021.792741 (PMC8666620; doi:10.3389/fphar.2021.792741)
Supplement: Supplementary file 1 [file Image2.pdf]

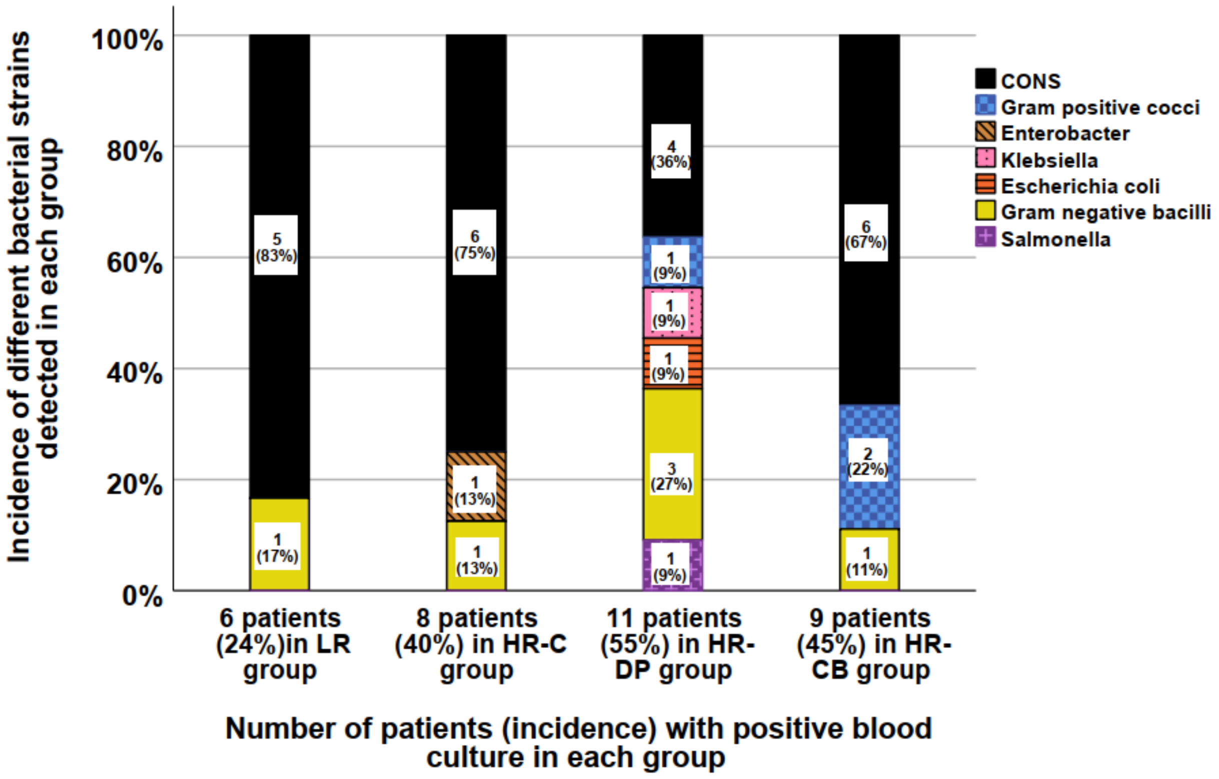

Supplementary Figure S2: The number of patients (incidence) with positive aerobic bacterial blood culture (obtained on day 6) in each group and the incidence of bacterial strains in each group.

LR: low risk for sepsis group, **HR-C:** high risk for sepsis control group, **HR-DP:** high risk for sepsis vitamin D and probiotics group, **HR-CB:** high risk for sepsis vitamin C and vitamin B1 group. **CONS:** Coagulase negative *Staphylococcus aureus*. **Data are number (incidence).**
