# Supplementary material for: Effect of Anti-Inflammatory and Antimicrobial Cosupplementations on Sepsis Prevention in Critically Ill Trauma Patients at High Risk for Sepsis
Source: Front Pharmacol. 2021 Nov 29;12:792741. doi: 10.3389/fphar.2021.792741 (PMC8666620; doi:10.3389/fphar.2021.792741)
Supplement: Supplementary file 2 [file Image1.pdf]

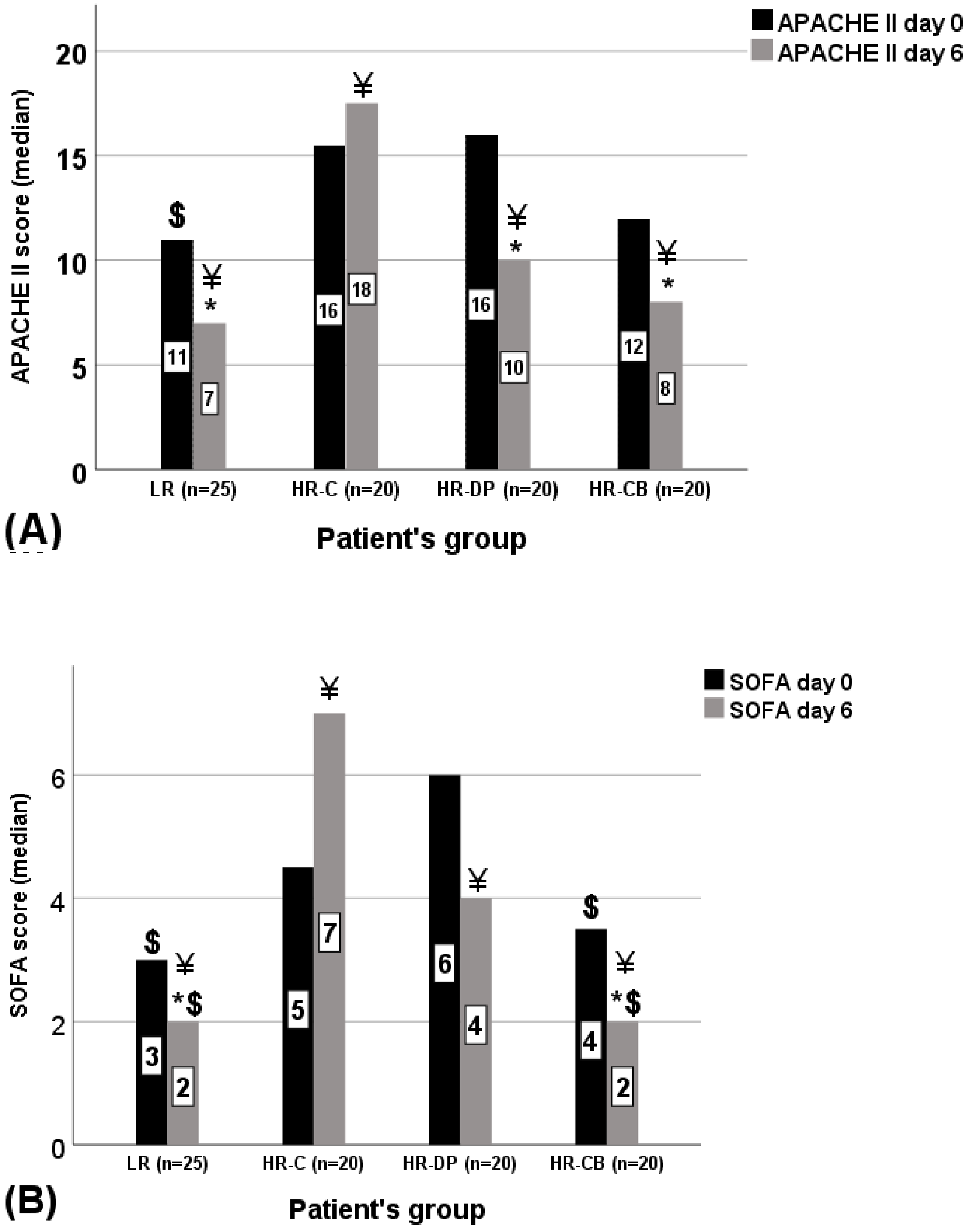

Supplementary Figure S1: APACHE II and SOFA scores on day 0 and day 6 in the tested groups, **(A):** APACHE II score on day 0 and day 6, **(B):** SOFA score on day 0 and day 6

LR: low risk for sepsis group, **HR-C:** high risk for sepsis control group, **HR-DP:** high risk for sepsis vitamin D and probiotics group, **HR-CB:** high risk for sepsis vitamin C and vitamin B1 group, **APACHE II:** Acute Physiologic Assessment and Chronic Health Evaluation score second version. **SOFA:** Sequential Organ Failure Assessment score. **Data are median (interquartile range)**. ***** = Significant with HR-C, **$** = significant with HR-DP. **¥** = Significant difference between day 0 and day 6 score within the same group. Significance level at **$p \leq 0.05$**
